# Enhancer engagement sustains oncogenic transformation and progression of B-cell precursor acute lymphoblastic leukemia

**DOI:** 10.1186/s13046-024-03075-y

**Published:** 2024-06-27

**Authors:** Giacomo Corleone, Cristina Sorino, Matteo Caforio, Stefano Di Giovenale, Francesca De Nicola, Frauke Goeman, Valentina Bertaina, Angela Pitisci, Clelia Cortile, Franco Locatelli, Valentina Folgiero, Maurizio Fanciulli

**Affiliations:** 1 IRCCS Regina Elena National Cancer Institute, Via Chianesi 53, Rome, 00144 Italy; 2 Department of Pediatric Hematology-Oncology and Cell and Gene Therapy, IRCCS Bambino Gesù Children’s Hospital, Viale Di San Paolo 12, Rome, 00146 Italy; 3 Department of Computer, Control, and Management, Engineering Antonio Ruberti, Sapienza University of Rome, Rome, 00161 Italy; 4 Department of Life Sciences and Public Health, Catholic University of the Sacred Heart, Rome, Italy

**Keywords:** Cis-regulatory elements, BCP-ALL, CRISPR/Cas-9, MYB, DCTD

## Abstract

**Background:**

Enhancer reprogramming plays a significant role in the heterogeneity of cancer. However, we have limited knowledge about the impact of chromatin remodeling in B-Cell Precursor Acute Lymphoblastic Leukemia (BCP-ALL) patients, and how it affects tumorigenesis and drug response. Our research focuses on investigating the role of enhancers in sustaining oncogenic transformation in children with BCP-ALL.

**Methods:**

We used ATAC-seq to study the accessibility of chromatin in pediatric BCP-ALL at three different stages—onset, remission, and relapse. Using a combination of computational and experimental methods, we were able to analyze the accessibility landscape and focus on the most significant cis-regulatory sites. These sites were then functionally validated through the use of Promoter capture Hi-C in a primary cell line model called LAL-B, followed by RNA-seq and genomic deletion of target sites using CRISPR-Cas9 editing.

**Results:**

We found that enhancer activity changes during cancer progression and is mediated by the production of enhancer RNAs (eRNAs). CRISPR-Cas9-mediated validation of previously unknown eRNA productive enhancers demonstrated their capability to control the oncogenic activities of the MYB and DCTD genes.

**Conclusions:**

Our findings directly support the notion that productive enhancer engagement is a crucial determinant of the BCP-ALL and highlight the potential of enhancers as therapeutic targets in pediatric BCP-ALL.

**Supplementary Information:**

The online version contains supplementary material available at 10.1186/s13046-024-03075-y.

## Background

Cancer treatment is challenged by intra- and inter-tumor heterogeneity, which calls for research into mechanisms that can act across patient characteristics to develop innovative therapies. While oncogenic potential for proliferation and dissemination is generally thought to be supported by a series of genetic events [1], recent studies have shown that the genetic profile alone does not fully explain cell phenotypes [2, 3]. Indeed, driver-coding mutations are rarely different in metastatic samples despite significant clinical and morphological differences from primary tumors [1, 4]. These findings highlight the importance of considering epigenetic changes in the development and progression of cancer, as they may play a crucial role in shaping tumor heterogeneity and response to therapy. Non-genetic events such as epigenetic reprogramming contribute to the phenotypic heterogeneity of cancer as much as genetic variation [1, 5], making it imperative to investigate these mechanisms.

B-cell precursor acute lymphoblastic leukemia (BCP-ALL) is a common childhood malignancy with a high cure rate, but relapse occurs in about 10% of patients [6]. Current risk stratification schemes consider factors such as time to relapse, relapse site, immunophenotype, and response to reinduction therapy. Despite the identification of novel BCP-ALL subtypes through large genomic analyses [7, 8], patient outcomes remain heterogeneous, and genetic alterations and transcriptomic shifts do not fully explain this variability. Therefore, investigating non-genetic mechanisms that contribute to BCP-ALL progression and response to therapy is essential for the development of effective treatments for all patients.

Little is known about the chromatin remodeling of BCP-ALL patients and the relative impact on tumorigenesis and drug response. One possibility is that chromatin plasticity affects the enhancer activity and engagement of TFs, thus supporting oncogenic pathways. Moreover, comparing chromatin openness in primary, remission, and relapse samples may provide insights into the biology of arising and response to cancer therapy [9].

Our study presents a comprehensive analysis of the chromatin accessibility landscape in pediatric BCP-ALL at different stages of the disease. We utilized advanced sequencing techniques to construct a detailed map of chromatin accessibility in BCP-ALL, which we further validated using a human primary cell line model and patients profiled with multi-omics (RNA-ATAC) single-cell technology. By linking enhancer activity to key target genes, we identified critical players in the transformation and progression of BCP-ALL. Our results were consistent across a large dataset of approximately 1200 patient transcriptomes, indicating that the transcriptional output of our identified target genes is independent of disease subtype. Furthermore, we demonstrated the direct and effective control of individual gene target expression and protein production using CRISPR-Cas-9 editing and provided further mechanistic evidence of the primary importance of RUNX and ERG transcription factors in the BCP-ALL context. Our findings highlight the critical role of enhancers in the development and evolution of BCP-ALL and offer the potential for developing innovative therapeutic strategies targeting these regulatory elements.

## Methods

### Patients’ characteristics

Patients newly diagnosed or relapsed BCP‐ALL were treated at the IRCCS Bambino Gesù Children's Hospital (Rome, IT). The immunophenotype was examined using multiparametric flow cytometry. Bone marrow (BM) was collected in test tubes containing sodium citrate as an anticoagulant and analyzed within 24 h of collection to determine the percentage of blasts. For each sample, 2 × 10^6^ cells were incubated for 15 min at room temperature in the dark, in the presence of monoclonal antibodies. Subsequently, each sample was subjected to osmotic lysis to eliminate contaminating red blood cells using 2 mL of a commercial ammonium chloride solution (150 mM NH4Cl, 10 mM NaHCO3 pH 7·4, BD Pharma LyseTM, BD Biosciences, San Diego, CA, USA) for 10 min at room temperature. The labeled cell suspension was then centrifuged at 400 g for 5 min at room temperature to separate the lysed red blood cell supernatant from labeled white blood cells. The supernatant was then removed, and the labeled cell pellet was resuspended in 200 μL of phosphate-buffered saline (PBS) to be immediately acquired using a DxFlex Beckman Coulter (Milano, Italy).

To reach sufficient cells analyzed and to obtain statistical significance, at least 1 × 10^6^ events/cells were recorded for each sample. MRD analysis was performed to determine the blast population using a Beckman Coulter DxFlex Flow Cytometer and analyzed using CytExpert Software (Beckman Coulter) according to the different analysis strategies.

Duraclone RE ALB tubes (Beckman Coulter) were used to perform the MRD analysis of BCP-ALL. Briefly, anti-CD19, anti-CD20, anti-CD38, anti-CD34, anti-CD45, anti-CD58, and anti-CD10 monoclonal antibodies were used to differentiate blasts from normal B lymphocytes. Immunophenotyping by flow cytometry is an integral part of the initial work-up of ALL patients because of its direct consequences for stratification and therapy. Flow cytometric analysis and interpretation/reporting of the findings should be performed according to the AIEOP-BFM ALL Immunophenotyping Consensus Guidelines.

### Relapse

The diagnosis of relapse can only be made if complete remission has been achieved.

Definitions:

Isolated bone marrow relapse:

- ≥ 25% lymphoblasts in the bone marrow without extramedullary involvement.

-  ≥ 5% and < 25% lymphoblasts in the bone marrow and confirmation of prior clonal abnormality by flow cytometry and/or cytogenetics/FISH and/or PCR.

Level of detection considered confirmatory (two methods at least):

-  ≥ 5% by flow cytometry.
-  > detection limit for FISH
- At least two aberrant metaphases for cytogenetics
- MRD increases by at least one log (true value) to ≥ 1% (≥ 1 × 10–2) by ASO RQ-PCR)

If only one confirmatory test is available, two consecutive time points are needed (typically at least one week apart).

Combined bone marrow relapse:

-  ≥ 5% lymphoblasts in the bone marrow and at least one extramedullary site *Complete Remission*

Complete remission can, per definition, not be stated before day 33 of the protocol.

Complete Remission (CR) has been achieved when the following criteria are fulfilled:

-  < 5% blast cells (M1) in the representative bone marrow with sufficient cellularity and signs of regeneration of normal myelopoiesis
-  ≤ 5 nucleated cells/µL in CSF or > 5 nucleated cells/µL, and no evidence of blasts in cytospin
- No evidence of leukemic infiltrates as evaluated clinically and by imaging; a preexisting mediastinal mass must have decreased by at least 1/3 of the initial tumor volume; identification of residual blast cells by PCR or flow cytometry is not decisive for the assessment of complete remission.

The “newly diagnosed” group consisted of 26 patients (4 matched patients included), 19 males (73%), and 7 females (27%), with a median age at diagnosis of 7,5 years (range 0,4–18). The group of relapsed patients included eight patients, seven males (88%) and one female (12%), with a median age at diagnosis of 8,05 years (range 2–18) and a median age at relapse of 10.6 (range 4–23). BMs, used as a negative control, were obtained from age-matched or adult healthy donors (HBM) who donated BM for transplantation at Bambino Gesù Children's Hospital.

### CD19-sorting selection

BMs derived from healthy donors and patients with BCP-ALL were sorted by CD19 expression to select the B-cell compartment and blast cells, respectively. Briefly, whole BM samples were incubated with Human B RosetteSep (Stemcell Technologies, CAN), following the manufacturer's instructions.

### Cell lines, transfections, and reagents

For immortalization, LAL-B cells were obtained from BCP-ALL bone marrow mononuclear cells infected with Epstein-Barr virus [10]. NALM-6 cells were bought from ATCC (CRL-3273), and the NALM-18 cell line was kindly provided by Dr Pende D. (IRCCS San Martino, Genova, Italy). All cell lines were cultured in RPMI-1640 medium (Euroclone) supplemented with 10% FBS (Thermo Fisher Scientific), 2 mM glutamine (Thermo Fisher Scientific), and 40 µg/ml gentamicin, and were cultured at 37 °C in a humidified atmosphere containing 5% CO_2_. Mycoplasma contamination was periodically checked by polymerase chain reaction (PCR) using the following primers:

- Forward: 5’ –ACTCCTACGGGAGGCAGCAGTA- 3’.
- Reverse: 5’ –TCGACCATCTGTCACTCTGTTAAC- 3’.

Nucleofection experiments with LAL-B cells were performed according to the manufacturer’s instructions using the Amaxa 4D-Nucleofector X kit L (Lonza). Cells were analysed 36 h after nucleofection using western blotting (WB) or quantitative real-time PCR (qRT-PCR).

### Promoter capture Hi-C

Hi-C in the LAL-B cell line was performed using an Arima-HiC Kit according to the manufacturer’s instructions. Briefly, 1 × 10^6^ cells were crosslinked with 1% formaldehyde, digested with a restriction enzyme cocktail, end-labeled with Biotin-14-dATP, and then ligated. The ligated chromatin was reverse-cross-linked and sonicated using a Bioruptor ultrasonicator to produce 300–500 bp fragments. Fragmented DNA was then size-selected to have a size distribution between 200–600 bp, and finally subjected to biotin enrichment. DNA libraries were prepared using an Accel-NGS 2S Plus DNA Library Kit ( Cat. No. 21024), and the resulting libraries were amplified using a KAPA Library Amplification Kit. Subsequently, the libraries were hybridized to specific SureSelect XT Human capture libraries (Agilent Technologies) and sequenced in paired-end mode (2 × 75 bp) using NextSeq 500 (Illumina, CA).

### RNA-sequencing

Total RNA was extracted from patient samples using Qiazol (Qiagen, IT), purified from DNA contamination through a DNase I (Qiagen, IT) digestion step, and further enriched by Qiagen RNeasy columns for gene expression profiling (Qiagen, IT). The quantity and integrity of the extracted RNA were assessed using a NanoDrop Spectrophotometer (NanoDrop Technologies, DE) and an Agilent 2100 Bioanalyzer (Agilent Technologies, CA), respectively. RNA libraries were generated using the same amount of RNA for each sample according to the Illumina TruSeq Stranded Total RNA kit with an initial ribosomal depletion step using Ribo Zero Gold (Illumina, CA, USA). The libraries were quantified by qPCR and sequenced in paired-end mode (2 × 75 bp) using NextSeq 500 (Illumina, CA, USA). For each sample generated by the Illumina platform, a pre-processing step for quality control was performed to assess sequence data quality and discard low-quality reads.

### ATAC-sequencing

To profile open chromatin, we used the ATAC-seq protocol developed by Buenrostro et al. [11], with minor modifications. B-cells were isolated from the malignant and control bone marrow aspirates. A total of 50,000 cells were washed once with 1X PBS and centrifuged at 500 g for 5 min at 4 °C. The cell pellet was lysed in ice-cold lysis buffer (10 mM Tris–HCl pH 7.4, 10 mM NaCl, 3 mM MgCl_2_, and 0.1% IGEPAL CA-630) to isolate the nuclei. If the cell pellet was flash-frozen at -80 °C, the morphology of the isolated nuclei was carefully inspected for integrity by trypan blue staining. The nuclei were centrifuged at 500 g for 5 min at 4 °C and subsequently resuspended on ice in 50 μl transposase reaction buffer containing 2.5 μl of Tn5 transposase and 25 μl 2xTD buffer (Nextera DNA Sample Preparation Kit from Illumina). After incubation at 37 °C for 30 min, the samples were purified using a MiniElute PCR Purification Kit (Qiagen) and eluting in 10 µl of elution buffer (10 mM Tris–HCl pH 8). To amplify transposed DNA fragments, we used NEBNext High-Fidelity 2 × PCR Master Mix (New England Labs) and Customized Nextera PCR Primers. Libraries were purified by adding Agencourt Ampure XP (Beckman) magnetic beads (1:1 ratio) to remove remaining adapters (left-side selection) and double-purified (1:0.5 and 1:1.15 ratio) for right-side selection. Libraries were controlled using a high-sensitivity DNA kit on a bioanalyzer (Agilent Technologies). Each library was then paired-end sequenced (2 × 75 bp) using a NextSeq 500 instrument (Illumina).

### Multi-omics single cell

Sorted B-cells were subjected to a nuclei isolation protocol. The nuclei were immediately processed with a 10 × Genomics Chromium controller using a Chromium Next GEM Single Cell Multiome ATAC + Gene Expression Kit. 5.000 cells with 90% viability were loaded for each sample. The libraries were generated according to the Chromium Next GEM Single Cell Multiome ATAC + Gene Expression protocol and the quality was checked using Agilent High Sensitivity DNA 1000 Kit on Agilent Tape Station. Libraries were quantified using a dsDNA High-Sensitivity (HS) Assay Kit (Invitrogen) on a Qubit fluorometer and the qPCR-based KAPA quantification kit. RNA libraries were sequenced on an Illumina Nova-Seq 6000 with 18.10.10.90 paired-end format and ATAC libraries with 50.8.24.49 paired-end format.

### Cell growth assay

Cell counts were performed by Countess Automated Cell Counter (Thermo Fisher Scientific, IT). For tetrahydrouridine (THU) (Calbiochem, cat. N° 584,222) sensitivity assay, NALM-6, NALM-18 and ALL-B cells were seeded at 300,000 cells/well in triplicate, treated with 10 µM, and analyzed at different time points (24h, 48h, 72h).

### Clonality and Penetrance index scoring strategy and relative analysis

The Clonality (CI) and penetrance index (PI) were calculated using two independent analysis workflows and were ultimately assigned to each genomic region included in the master list of accessibility (see supplementary data). CI is a patient-specific standardized metric determined by the following formula calculated for the peak repertoire of each sample independently: Nscore = ((peak read count/peak size)⋅10^–6^)* 10^–3^ /total mapped reads. The peak read count was assessed using the multicov function of bedtools. Then, the peaks in each sample were arranged from the highest to the lowest Nscore. The ordered list of N scores was divided into percentiles ranging from 1 (highest enrichment) to 100 (lowest enrichment). All peaks present in the master list and absent in the given sample had an assigned CI of 0. The PI was assigned to each peak of the master list and represented the number of patients sharing each peak in the patient cohort. At each peak, the PI value ranged from 1 (only one patient carrying the given peak) to 32 (the total number of patients carrying the given peak).

### Statistics

Statistical analyses were performed using the R programming language. Details of the statistical methods used are provided in the figure legends and results sections. Detailed descriptions of the computational analyses are available in Supplemental Methods.

### Competing interest

The authors declare no competing financial interest.

## Results

### Genome-wide mapping of chromatin accessibility in a longitudinal cohort of pediatric BCP-ALL defines the disease stage

**Fig. 1:**
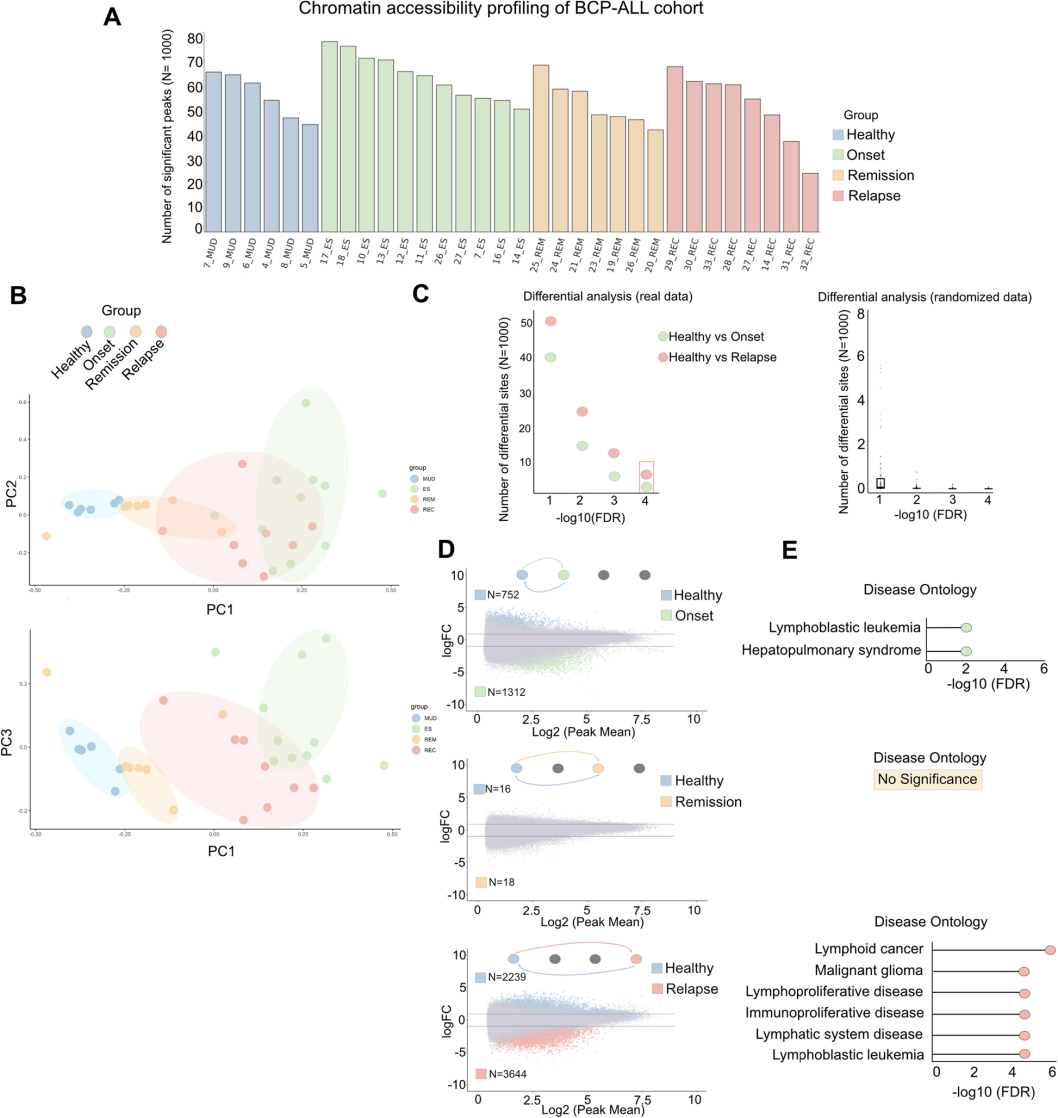
Differential analysis of BCP-ALL accessibility during cancer progression. **A** Histogram showing the total number of ATAC-seq significant peaks x sample profiled. X-axis: name of the sample; Y-axis: Absolute number of significant peaks. Color legend: Blue = Healthy samples; Green = Samples at Onset; Orange = Samples at Remission; Relapse = Samples at relapse. **B** PCA of accessibility profiles of our patient cohort. Up: PCA between principal component 1 (x-axis) and principal component 2 (y-axis). Down: PCA between principal component 1 (x-axis) and principal component 3 (y-axis). Shaded areas in the PCA plot represent 90% confidence ellipses. Color legend: Blue = Healthy samples; Green = Samples at Onset; Orange = Samples at Remission; Relapse = Samples at relapse. **C** Left: Differential analyses of accessibility profiles between Healthy vs. Onset (green point) and Healthy vs. Relapse (red point). X-axis: different points of significance; Y-axis: Number of differential peaks identified in the analyses. Right: Differential analyses were performed by applying 100 random sampling from the patient cohort. Group sizes matched the Healthy, Onset, and Relapse cohort. X-axis: different points of significance; Y-axis: Number of differential peaks identified in the analyses. **D** MA plot of the differential peak accessibility. Top: Healthy vs. Onset; Middle: Healthy vs. Remission; Bottom: Healthy vs. Relapse. X-axis: Log2(Peak mean), Y-axis: Log Fold Change of the differential accessibility of peaks. N = number of significant differential peaks identified in the analysis where logFC > 0.7 is upregulation in Healthy; logFC < -0.7 is upregulation respectively at onset (green), remission (orange), and Relapse (red). **E** Disease ontology associated with the differential analysis of accessibility. Top: upregulation at the Onset; Middle: upregulation at the Remission; Bottom: upregulation at the Relapse. Analysis was performed against Healthy tissues. The analysis is performed with the GREAT tool

We designed the study to include 26 cases of BCP-ALL obtained at onset (*N* = 11), remission (*N* = 7), and relapse (*N* = 8) not matching. To identify the diversity of malignant B cells, we selected a control cohort of healthy bone marrow (HBM) from six adult donors who donated BM for transplantation. The patients were profiled for the most common molecular abnormalities in BCP-ALL using cytogenetics (Table 1). Interestingly, cytogenetics of patients at diagnosis did not show any diagnostic genetic abnormalities in 64% of patients, while this frequency dropped to 25% at relapse. We calibrated our study by collecting B cells from fresh BM samples, isolating CD19 + cells by immunodensity, and then profiling using ATAC-seq. These experiments revealed accessible sites among all the samples, ranging from ~ 20 k to ~ 80 k sites. This strategy produced a cumulative number of 150,123 chromatin-accessible sites (Fig. 1A). Approximately 20% of these sites mapped within 5 kb of the closest transcription starting sites (TSS) at loci putatively considered as promoters. Interestingly, all selected groups of patients shared the same proportion of promoter-like active sites (Fig. S1A). Notably, while healthy tissues were strongly defined by promoter-like activity, the onset group showed a linear increase in the number of active sites at distal loci (Fig. S1A). Furthermore, promoter activity was equally shared among our cohort (Figs. S1B-C), while the largest part of Cis-Regulatory Elements (CREs) was encoded at distal genomic loci, accounting for ~ 80% of the total number of sites. In addition, the onsets exhibited much stronger activity in the non-coding and intron regions than in the other groups (Fig. S1B).

**Table 1:** Patient cohort molecular abnormalities

Table 1	**Newly Diagnosed Patients**	**Relapsed Patients**			
	**N.or median (% or range**)	**N.or median (% or range)**			
Number Of Patients	30 (6 matched)	10			
Gender					
Females	9 (30%)	2 (20%)			
Males	21 (70%)	8 (80%)			
Age at diagnosis (years)	6,7 (0,4 - 18)	8,5 (2 - 18)			
Age at Relapse (years)	-	11,7 (5 - 23)			
Disease Status					
Diagnosis	30 (100%)	-			
1^ Relapse	-	4 (40%)			
2^ Relapse	-	5 (50%)			
3^ Relapse	-	1 (10%)			
Remission	7				
Molecular abnormalities					
None	15 (50%)	5 (50%)			
t(12;21) (TEL/AML1)	6 (20%)	2 (20%)			
t(9;22) (BCR/ABL)	3 (10%)	1 (10%)			
t(9;11)	1 (3,3%)	0 (0%)			
t(3;9)	1 (3,3%)	0 (0%)			
t(3;20)	1 (3,3%)	0 (0%)			
t(9;10)	1 (3,3%)	0 (0%)			
t(5;11)	1 (3,3%)	1 (12%)			
t(6;9)	1 (3,3%)	1 (12%)			
t(7;9)	1 (3,3%)	1 (12%)			
t(11;13)	1 (3,3%)	1 (12%)			
t(16;1)	1 (3,3%)	1 (12%)			
t(8;14)	1 (3,3%)	1 (12%)			
r(KMT2A)	3 (10%)	1 (12%)			
Aneuploidy					
Yes	9 (30%)	3 (30%)			
No	21 (70%)	7 (70%)			
*Defined as chromosome number > or < 46					
Matched patients: samples from the same patient at different stage of disease					
**Onset**	Gender	Age at diagnosis	Age at Relapse	Genetic riarrangment at onset	Aneuploidy
#1 (17_ES)	M	3		t(3;9); t(3;20)	Yes
#2 (13_ES)	F	2		N.D.	Yes
#3 (12_ES)	M	18		t(9;22)(BCR/ABL); t(9;10);	Yes
#4 (11_ES)	F	4		t(12;21)(TEL/AML1)	N.D.
#5 (26:_ES) *	M	4		N.D.	Yes
#6 (27_ES)**	M	4		t(12;21)(TEL/AML1)	N.D.
#7 (07_ES)	M	13		N.D.	N.D.
#8 (14_ES)***	M	13		N.D.	N.D.
#9 (18_ES)	F	8		N.D.	N.D.
#10 (10_ES)****	M	3		t(12;21)	N.D.
#11 (16_ES)	M	4		N.D.	Yes
#12 (E1)*****	F	3		r(KMT2A); (BCR/ABL); (TEL/AML1)	N.D.
#13 (E2)******	M	3		Plurirearrangment	N.D.
**Remission**					
#14 (19_REM)	M	1		N.D.	N.D.
#15 (24_REM)	F	9		N.D.	Yes
#16 (25_REM)	M	12		r(KMT2A)	N.D.
#17 (21_REM)	F	8		N.D.	N.D.
#18 (23_REM)	M	6		t(12;21)(TEL/AML1)	N.D.
#19 (20_REM)	F	3 mesi		t(9;11)	N.D.
#20 (26_REM)*	M	4		N.D.	N.D.
**Relapse**					
#21 (28_REC)****	M	3	6	t(12;21)(TEL/AML1)	N.D.
#22 (33_REC)	F	18	23	t(9;22)(BCR/ABL); r(KMT2A)	Yes
#23 (27_REC)**	M	4	5	N.D.	N.D.
#24 (14_REC)***	M	13	15	N.D.	N.D.
#25 (31_REC)	M	13	15	t(5;11); t(6;9); t(7;9); t(11;13); t(16;1)	N.D.
#26 (32_REC)	M	5	14	N.D.	N.D.
#27 (29_REC)	M	15	20	t(8;14);	Yes
#28 (30_REC)	M	2	6	N.D.	Yes
#29 (R1)*****	F	3	7	N.D.	N.D.
#30 (R2)******	M	3	6	(TEL/AML1)	N.D.

*ND* Not Detected

*Matched samples

Next, we sought to investigate the relationship between the DNA accessibility landscape and BCP-ALL phenotype at different disease stages. Principal Component Analysis showed that healthy and remission patients were more homogeneous, whereas patients at onset and relapse were highly heterogeneous, thus confirming an involvement of regulatory regions in supporting distinct cancer phenotypes (Fig. 1B). Notably, we observed that even samples with a very low minimal residual disease exhibited differences in the accessibility landscape compared to those obtained from healthy individuals, suggesting that therapy may contribute to differentiation at the level of chromatin accessibility. Collectively, our analysis lends additional support to the concept that the regulatory repertoire plays a critical role in defining the phenotypic heterogeneity of BCP-ALL.

### Cis-regulatory activity is a main phenotypical determinant of BCP-ALL

CREs are distant regulatory elements that are positively associated with gene transcription and, thus, are key elements in determining cell phenotypes [12, 13]. To study the process of CREs modulation during BCP-ALL progression, we surveyed the variability of each 150,123 identified CRE by performing differential analysis among patients in the healthy, onset, remission, and relapse groups (Fig. 1C-D). We found evidence of highly diverse epigenetic profiles in onset and relapse, with at least tenfold more differentially active CREs than expected by chance (Fig. 1C, right). As previously observed in other cancer types [14], the prevalent differential patterns were exhibited between the healthy vs. onset and healthy vs. relapse groups (Fig. 1D and Figs. S1D). By applying a very stringent threshold of FDR < 10^–4^ of significance, we selected 1312 CRE sites upregulated at the onset, which were strikingly enriched with ontologies specifically associated with Lymphoblastic Leukemia (Fig. 1E). A total of 5883 sites were actively modulated, of which 3644 were upregulated during relapse (Fig. 1D, bottom). The interrogation of disease ontology showed a more robust enrichment of lymphoid cancer and lymphoblastic diseases, suggesting a post-therapy selection of CREs associated with the cancer phenotype (Fig. 1E, Figs. S1E-F). Overall, these data support the hypothesis that the pathogenesis of BCP-ALL is sustained by a differential engagement of CREs, which may play a relevant role in determining the behavior of cells following treatment.

### Dissection and tracking of cis-regulatory element activity during BCP-ALL evolution

**Fig. 2:**
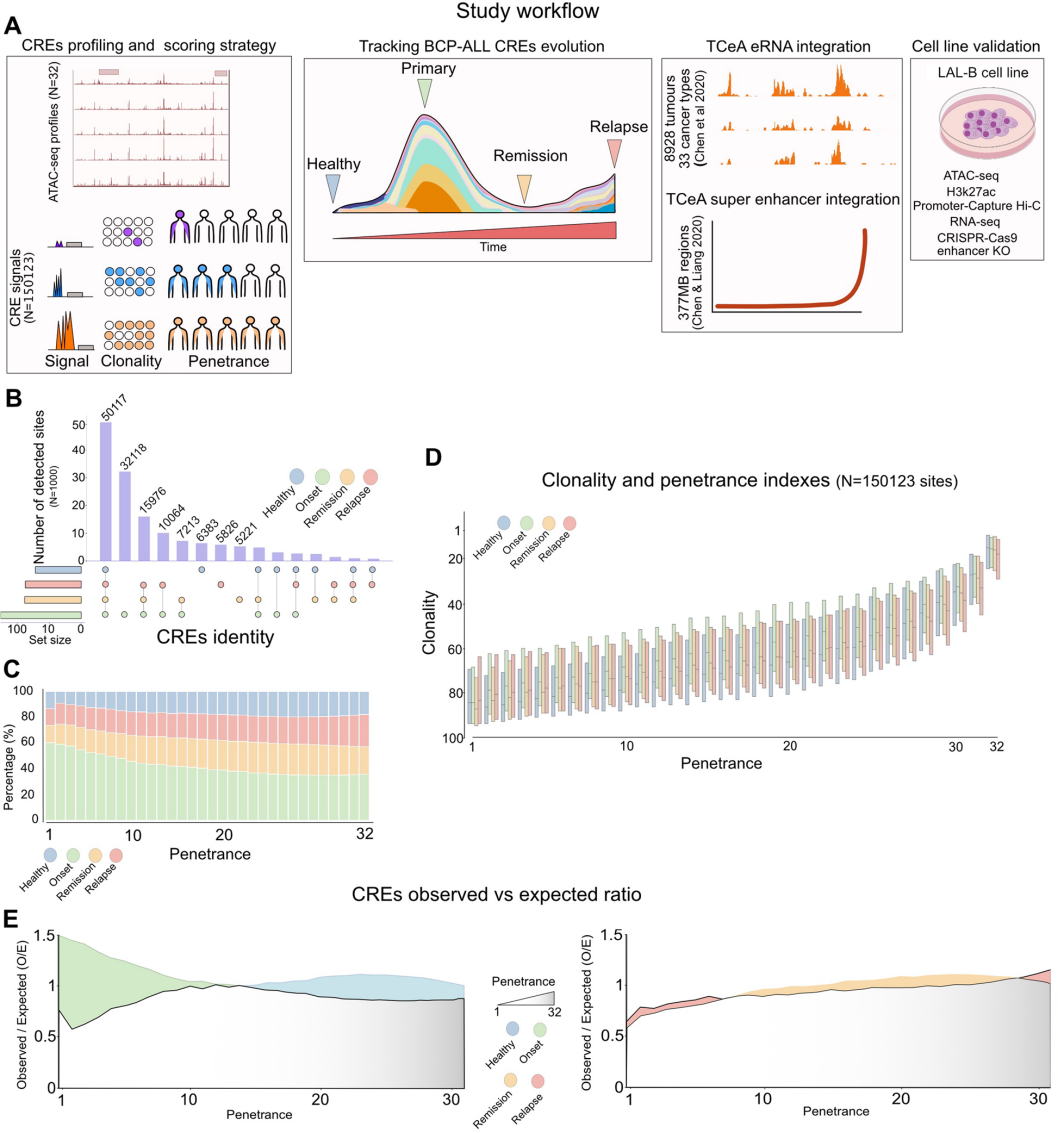
Dissection of cis-regulatory heterogeneity of BCP-ALL. **A** Workflow of the study. From left to right: We profiled 32 samples of BCP-ALL to identify putative cis-regulatory regions. With a scoring strategy based on the Clonality and Penetrance indices (see Supplementary methods), we dissected the accessibility landscape and prioritized the study toward the most clonal/penetrant cis-regulatory sites. Then, these two scores assigned to each CRE were used to monitor clonality and penetrance during BCP-ALL evolution by assessing the variation of CREs modulation at Healthy > Primary > Remission > Relapse stages. To provide more insights into the functional role of the selected CREs, we integrated data from the TCEA portal, which provides enhancer RNA-seq profiles from 8928 samples of 33 different cancers. Furthermore, we integrated a 377 MB region of super-enhancer into our selection. We validated several elements with CRISPR KO and experimental procedures among all the selected CRE sustaining BCP-ALL progression. **B** Upset plot of detected peaks among the different groups of patients. X-axis: Intersection combination; Y-axis: the absolute number of detected sites. Color legend: Blue = Healthy samples; Green = Samples at Onset; Orange = Samples at Remission; Relapse = Samples at relapse; Violet: Barchart of the number of detected sites at each intersection. **C** Left: Stacked bar chart representing the percentage of significant peaks (y-axis) x group in the function of the penetrance index (x-axis); Color legend: Blue = Healthy samples; Green = Samples at Onset; Orange = Samples at Remission; Relapse = Samples at relapse. **D** Boxplots show the median Clonality Index value and interquartile ranges for each detected peak x disease stage in the function of the Penetrance index. Color legend: Blue = Healthy samples; Green = Samples at Onset; Orange = Samples at Remission; Relapse = Samples at relapse. **E** Observed/Expected (O/E) ratio of peaks (y-axis) at any penetrance score (x-axis) between Healthy vs Onset (left) and Remission vs Relapse (right). Color legend: Blue = Healthy samples; Green = Samples at Onset; Orange = Samples at Remission; Relapse = Samples at relapse

Once determined the chromatin accessibility variability among BCP-ALL stages, we performed a more in-depth analysis using a combination of numerous experimental and computational techniques (Fig. 2A, Supplementary Methods) for dissecting each regulatory element. Unsurprisingly, the onset group had the highest number of activated CREs, accounting for more than 120,000 detected sites (Fig. 2B). Roughly 50 k CREs were shared among each disease stage, whereas 32,118 CREs were present only in the onset group. A subset of 32,118 sites was detected only at onset, and 5826 sites were specific to relapse (Fig. 2B). Among all the sites, 60% were detected as private or low penetrant in the onset group. This percentage decreased to 35% in the more penetrant regions of the cohort. In contrast, the healthy, remission, and relapse groups exhibited less than 10% of the detected sites in private or low shared regions, suggesting that the onset group is characterized by a larger plethora of distinct cell subclones sustained by polythetic activity of regulatory elements than the other groups (Fig. 2C).

To provide qualitative insights into the relative contribution of each detected CREs to the BCP-ALL phenotype, we applied a computational framework to dissect the regulatory heterogeneity of the chromatin accessibility landscape. We observed that penetrance and clonality (see Computational Methods) had a strong linear relationship in each sample group (Fig. 2D). These observations were further corroborated by linear regression analysis independently performed in each disease group, demonstrating a positive relationship between clonality and penetrance (Fig. S2A). Indeed, the onset group exhibited a higher observed heterogeneity than expected at low penetrance indices (PI = 1–14), while highly penetrant CREs were observed more in healthy samples than expected (Fig. 2E). In contrast, remission to relapse did not change significantly.

In summary, our findings provide strong evidence supporting the notion that the distinct engagement of regulatory elements may play a role in defining cancer stages. These observations highlight the dynamic nature of chromatin accessibility in cancer and suggest that the establishment and maintenance of a regulatory repertoire is a key factor in cancer progression and response to therapy.

### The regulatory landscape of BCP-ALL dynamically changes during cancer evolution

**Fig. 3:**
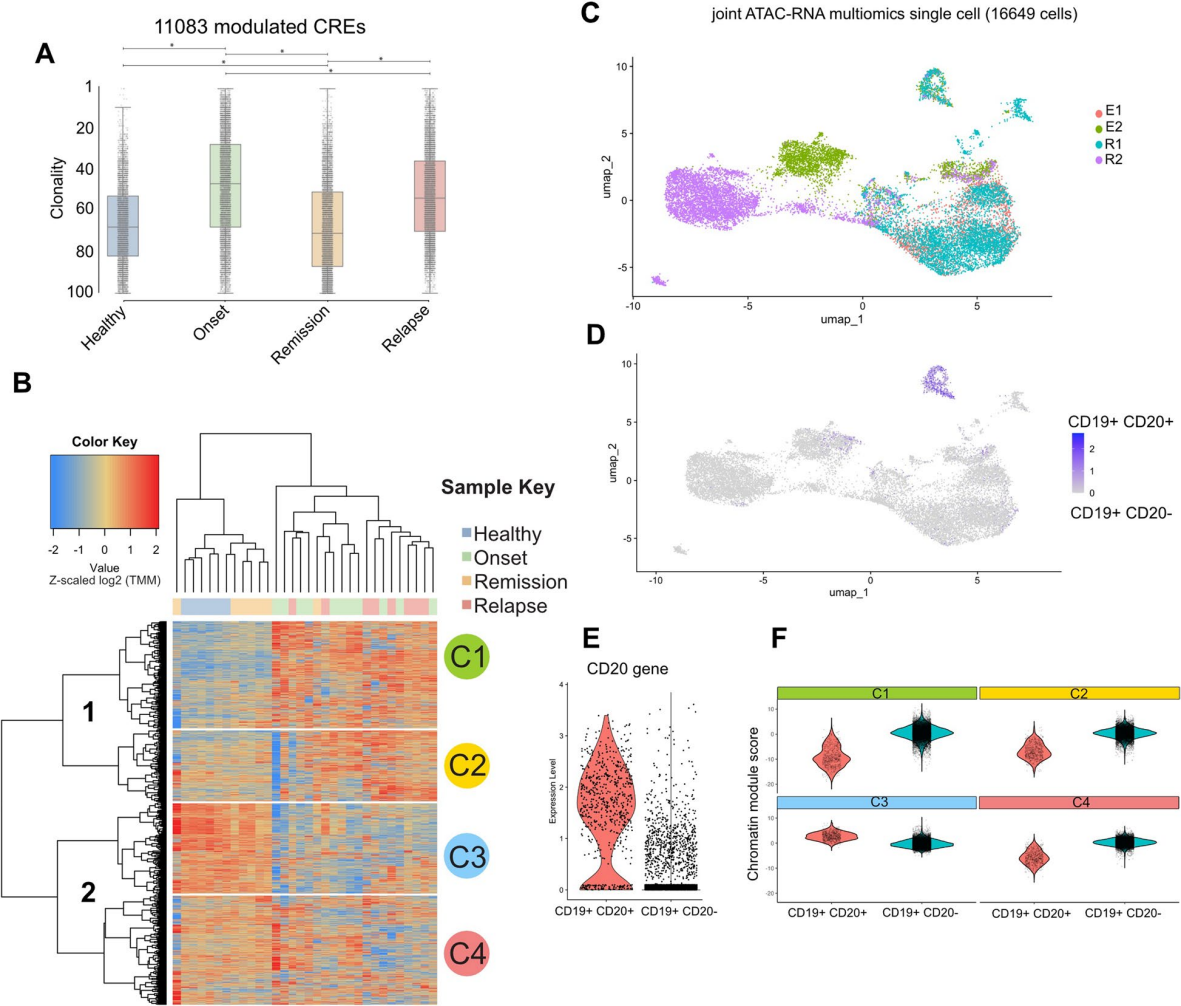
Enhancer engagement varies amongst BCP-ALL cancer stages. **A** Boxplot depicting the Clonality index of the selected CREs at the Healthy, Onset, Remission, and Relapse status. Color legend: Blue = Healthy samples; Green = Samples at Onset; Orange = Samples at Remission; Relapse = Samples at relapse. Statistical test: Kruskal–Wallis test followed by Dunn’s test. Pval = * < 10^–^4. Statistical significance was calculated using a pairwise, two-tailed t-test. **B** Unsupervised Clustering Heatmap showing z-scaled log2(TMM) score enrichment of the selected CREs (*N* =) in each given patient of the cohort. The analysis identified two main branches of data (left) and four main clusters named C1, C2, C3, and C4 (right). Color legend: Blue = Healthy samples; Green = Samples at Onset; Orange = Samples at Remission; Relapse = Samples at relapse. **C** UMAP of 16,649 CD19 + cells based on the integrated RNA and ATAC embedding. Cells are colored according to the sample they belong. Matched samples: E1-R1; E2-R2. (E = Onset; R = Relapse. **D** UMAP shows cells grouped into malignant (CD19 + CD20-) and non-malignant cells (CD19 + CD20 +). Color scheme representing the relative enrichment of the CD20 (MS4A1) gene expression. **E** Violin plot representing the normalized CD20 (MS4A1) gene expression divided according to the two populations detected. **F** Genomic regions belonging to the C1, C2, C3, and C4 were individually evaluated in the scATAC data. The resulting enrichment is represented in the violin plots divided according to the population. Y-axis: chromatin module score

We hypothesized that a subset of regulatory regions is dynamically engaged to drive onset and relapse. To test this hypothesis, we selected 11,083 highly penetrant regulatory sites that showed poor activity in healthy tissues, dynamically changing their relative clonality over time (Fig. 3A, Supplementary Table 2). In agreement with this, unsupervised clustering of normalized ATAC-seq enrichment at the selected CREs highlighted the similarity between the healthy and remission group profiles. The clustering identified two major clades, with one showing higher activity of CREs only at the onset and relapse (C1 and C2) (Fig. 3B). Notably, ~ 90% of the selected CREs were distal to the closest gene (Fig. S3A). Functional characterization demonstrated that our approach successfully targeted regulatory sites strongly involved in lymphocyte activation/differentiation and in sustaining the regulation of genes linked to lymphoblastic leukemia, lymphadenopathy, and, more generally, autoimmune diseases (Fig. S3B).

To validate our findings, we recruited 2 patients with BCP-ALL who had a matching onset and relapse. We sorted the BM for the CD19 + cells and used multi-omics single-cell sequencing (ATAC-RNA seq) to examine the cell type composition. By doing so, we captured a snapshot of the cells and translated the findings from bulk to single-cell level. After quality control, we obtained a total of 16,649 sequenced CD19 + cells. The results showed that both patients had independent clusters of cells at onset and relapse (as shown in Fig. 3C). There were 12 clusters of cells, and the ninth cluster (Fig. S3C) was found to be common among all the samples. This cluster was strongly classified by the MS4A1 gene (CD20) expression, which is a recent marker of non-leukemic, normal B-like state [15] (Fig. 3D-E). At single cell resolution, the chromatin accessibility levels of the C1:C4 cluster identified in the bulk cohort showed a similar behavior. Indeed, in malignant cells, C1 and C2 were strongly upregulated, while C3 was upregulated in healthy-like cells (Fig. 3F; Fig. S3E).

Taken together, these data show the identification of clusters of regulatory elements that are strongly upregulated only under progressive conditions, both at bulk and single-cell levels.

### Transcription factors and active enhancers are key elements of BCP-ALL progression

**Fig. 4:**
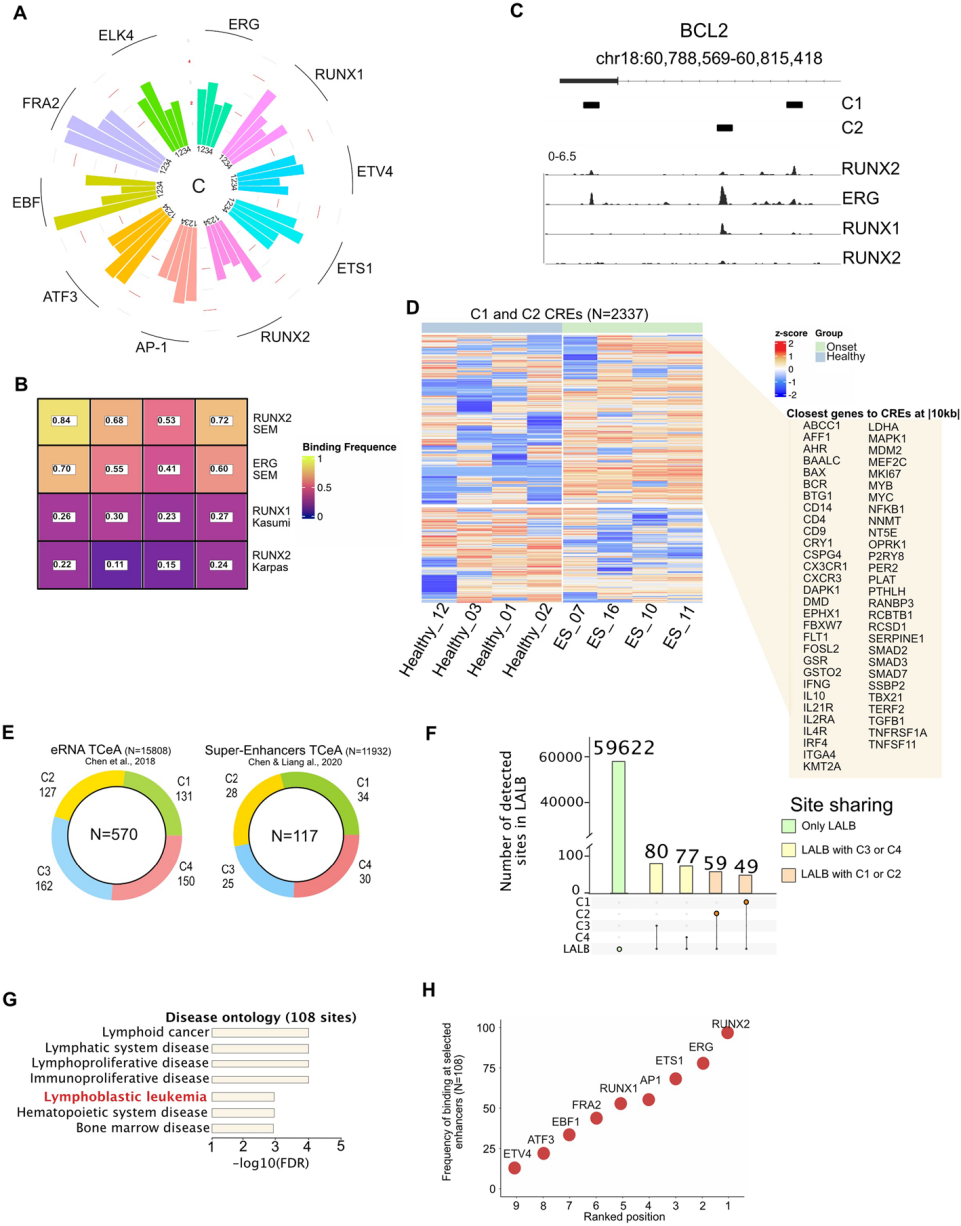
RUNX2, ERG binding together with eRNA production have a key role in CREs activity. **A** Polar bar plot depicting transcription factor motif enrichment at C1, C2, C3, and C4 sites. Bar plot representing the Observed/Expected ratio of the transcription factor motif at any given C cluster. **B** Heatmap showing the frequency of binding of RUNX2 and ERG from SEM cell lines (GSM3312807) and RUNX1 in Kasumi (GSM2026066) and Karpas (GSM4591424) cell lines. Color scheme from violet to yellow follows the frequency of C1, C2, C3,C4 sites bound by the selected transcription factors. **C** TFs binding at BCL2 locus. The ChIP-seq data binds the selected enhancers included in C1 and C2 (black boxes). **D** Unsupervised heatmap depicting eRNA at selected CREs (C1, C2) not matching exons annotation (hg19). RNA-seq data from the sample cohort composed 4 Healthy tissues and four tissues at the onset. Data were normalized and scaled with z-scoring. The window (right) highlights the closest genes (distance ranging |10 kb| from each CREs) associated with the upregulated regions at the onset. **E** Stacked pie chart of C1, C2, 3, C4 selected CREs intersected with ERNA TCeA portal (left) and Super-Enhancers TCeA portal (Right). Color legend: Green = C1; Yellow = C2; Blue = C3; Red = C4. **F** Upset plot of C1, C2, C3, and C4 selected CREs and LAL-B ATAC-seq peaks. X-axis: Intersection combination; Y-axis: the absolute number of detected sites at each intersection. Color legend showing the private site to only LAL-B (green), site shared amongst LAL-B and C3 or C4 (yellow), and site shared amongst LAL-B and C1 or C2 (orange) **G** Disease Ontology of upregulated CREs at the onset obtained with GREAT tool. **H** Dot plot shows the frequency (y-axis) of the Transcription Factors at the 108 selected CRE. Transcription factors are ranked by the number of binding events among the selected 108 CREs. Data obtained from ChIP-seq available at (see Table 1.)

To identify the mechanistic factors behind the activation of C1 and C2 clusters, we delved into the motifs of the regulatory elements and searched for possible transcription factor binding. We performed motif analysis and analyzed the observed/expected ratio of the most significant elements in the first clade (C1 and C2 clusters). Interestingly, we found that well-known B cell development drivers with established oncogenic potential were putatively binding to the selected CREs. Previous studies have demonstrated that aberrant modulation of EBF1, ETS1, ERG, and RUNX transcription factors has significant effects on lymphoid neoplasms derived from B cell progenitors [16, 17] (Fig. 4A). To further investigate the regulatory landscape in BCP-ALL, we integrated the analysis of TF ChIP-seq data from leukemic cell lines, demonstrating the fundamental engagement of key regulatory elements of hematopoiesis, RUNX2 and ERG [18, 19], at the selected CREs (Fig. 4B-C).

Functional characterization of a specific CRE can be a challenging task [19]. To address this, we focused on CREs that produced RNA (known as eRNA) in patients, as this has been shown to be indicative of their functionality. To identify these eRNA-producing CREs, we performed RNA-seq on 4 healthy and 4 onset patient samples and measured eRNA productivity at the previously identified C1 and C2 CRE loci. Unsupervised hierarchical clustering analysis revealed that 1092 CREs were significantly more productive in the onset samples compared to healthy samples (Fig. 4D), providing important insights into their potential functions in the context of cancer. We then linked these highly productive CREs to their closest gene. Interestingly, the selected CREs were found to potentially regulate key BCP-ALL phenotype determinants, such as ERG, KMT2A, and MYB. Thus, our approach offers a promising strategy for identifying CREs that may play a critical role in the regulation of cancer-specific gene expression programs [20, 21]. To build a more accurate classification of the selected productive CREs, we integrated our analysis with pre-annotated productive enhancers and super-enhancers [22] together with the accessibility landscape of LAL-B, a primary cell line generated by manipulation of BM from the patient at BCP-ALL onset [10]. Accordingly, 570 enhancers and 117 super-enhancers were selected among the C1, C2, C3, and C4 clusters (Fig. 4E). In addition, the ATAC-seq peaks of the eRNA productive sites in each sample of our cohort were highly heterogeneous at onset and were generally more clonal (higher RI) (Fig.S3F). We then compared the LAL-B accessibility profiles with those of the patient cohort (Fig. S3G) observing 108 active CREs shared with C1 and C2 (Fig. 4F) and linked to key determinants of lymphoid and bone marrow neoplasms, including lymphoblastic leukemia (FDR < 10^–3^) (Fig. 4G). Notably, ChIP-seq data of lymphoid cancers, including BCP-ALL, showed that RUNX2, ERG, and ETS1 (Supplementary Material 4) bind to over 70% of the selected 108 loci (Fig. 4H). Then, we screened the ENCODE cell line profiles of H3K27ac at the 108 selected loci (Fig. S4A), observing extensive engagement of enhancer activity that is not specifically lymphoid-dependent. Interestingly, the majority of identified enhancers have never been detected in previous studies. These findings suggest that active RNA productive enhancers, regulated by ERG and RUNX transcription factors, may play a significant role in determining the BCP-ALL phenotype.

### Long-range chromatin interactions add functional insights into BCP-ALL primary cell line

**Fig. 5:**
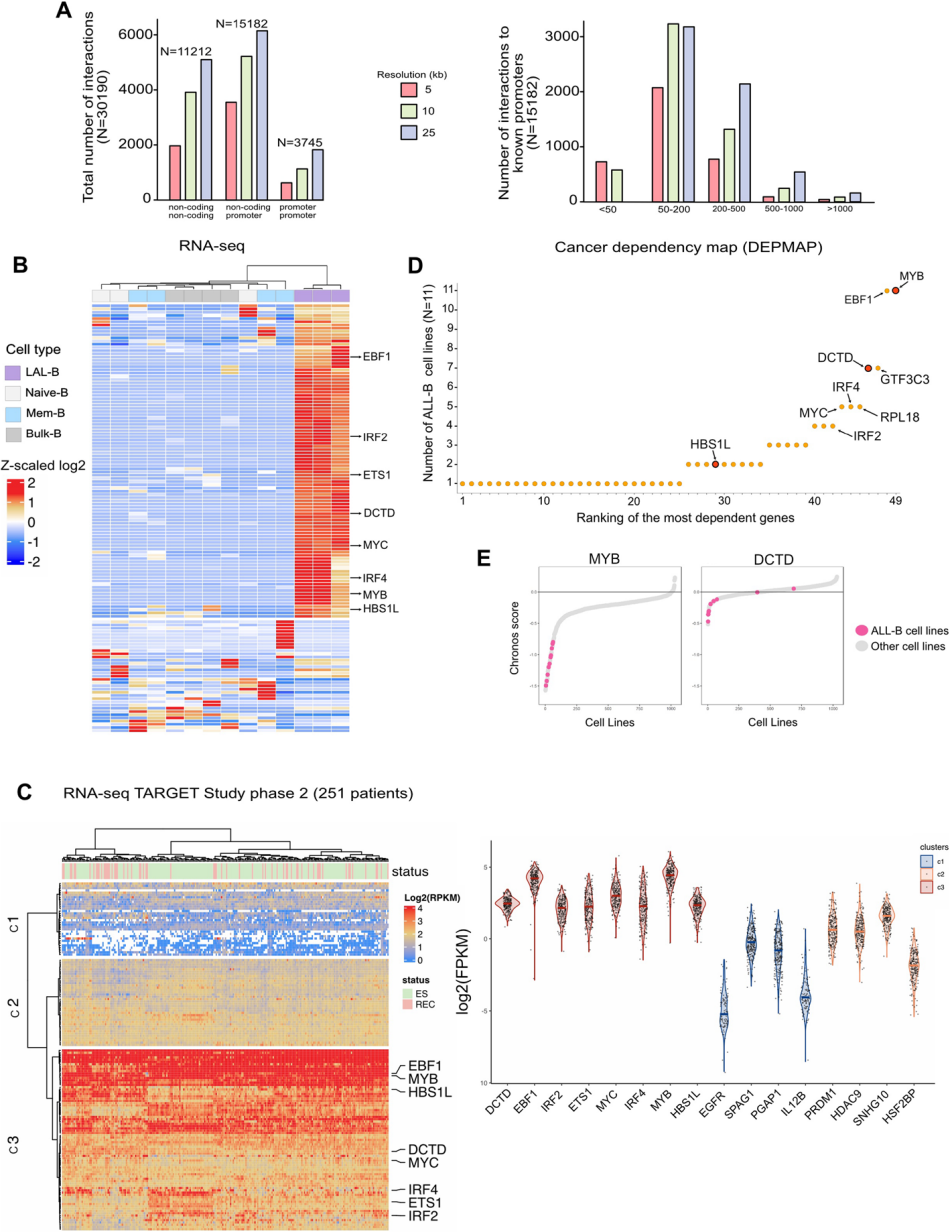
Identification of enhancer-target genes in LAL-B. **A** Barchart summarizes the number of absolute significant interactions of Promoter-Capture HiC in LAL-B cells grouped by annotation of anchor and target in the genomic context (left) and the number of absolute interactions specific to only CRE-Promoters (hg19) in LAL-B grouped by range distance (right). Color legend: Red = 5 kb resolution; Green = 10 kb resolution; Violet = 25 kb resolution. **B** Unsupervised clustering of gene expression (RNA-seq) of LAL-B, Naïve-B, Mem-B, and Bulk-B cells (triplicates for each category) obtained from Calderon et al. Nat. Gen 2019. Data were normalized and scaled with z-scoring. Genes interrogated in the heatmap (*N* =) are selected by evidence of looping with the 108 selected CREs. Color legend: Violet = LAL-B cells; Light grey = Naïve-B; Blu = Mem-B; Grey = Bulk-B cells. **C** *Left:* Unsupervised clustering of 251 patient transcriptome available atphs000218. The clustering identified C1, C2, C3 clusters. Color scheme based on the relative gene log2(FPKM). Red: transcriptional active genes; Blue: inactive genes; Yellow: low activity level. *Right:* Violin plots depicting the relative transcriptional output of a selected list of genes. Dots represent patients from the figure on the left. The color scheme is according to the three different clusters (C1, C2, C3). "*The results published here are in whole or part based upon data generated by the Therapeutically Applicable Research to Generate Effective Treatments (*https://ocg.cancer.gov/programs/target*) initiative, phs000218. The data used for this analysis are available at *https://portal.gdc.cancer.gov/projects*."* **D** Plot showing the ranked most dependent genes of BCP-ALL among the selected from Fig. 4B (x-axis) in the function of the number of ALL-B cell lines (*N* = 11) ranking at the top 10% of the most sensitive cell lines among the total number of available cell lines in the DEPMAP portal at any given gene. **E** Plots showing the Chronos score of MYB and DCTD genes of the selected ALL-B cell lines (pink) and all the other cell lines (grey). The highlighted ALL-B cell lines are the following: 697, JM1, SEM, RCHACV, NALM6, REH,ROS50, SEMK2, HB1119, NALM16, P30OHK

Predicting enhancer-gene interactions in a given cell type context lacks general rules that can be uniformly applied [23]. Therefore, our primary effort was to univocally identify the genes regulated by the selected enhancers. To address the functional role of the selected CREs, we first profiled the chromatin-interacting landscape of LAL-B cells using in situ Promoter capture HiC [24]. This analysis revealed short- and long-range interactions by analyzing the data at three different map resolutions (5 kb, 10 kb, 25 kb) (Fig. 5A). Our analysis detected 30,190 genomic interactions, of which ~ 15 k were classified as promoter–CRE looping. Approximately 11 k loops were detected between two non-promoter CREs, while 3745 loops were observed between the two known promoters (Fig. 5A, left). Nevertheless, we identified only ~ 1000 interactions at distances shorter than 50 kb (Fig. 5A, right) and noticed the complexity of the interactions between a given promoter and numerous CREs that interact with each other (Figs. S5 and S6A). Although recent data confirm that enhancer gene-target prediction can be inferred simply by gene proximity at high precision [25], we integrated our Promoter-capture HiC approach with the ATAC-seq results from patients and the LAL-B cell line (the selected 108 enhancers), and CTCF and Pol2 ChIA-Pet of the K562 cell line to survey every possible enhancer-gene contact (Supplementary material 4: Table 2). We measured the transcriptional and accessibility outputs of selected CREs and their relative gene targets in LAL-B cells and lymphoid cells at three stages of differentiation (naïve B cells, mem-B cells, and plasmablasts) obtained from healthy individuals [26]. We found that out of 108 selected enhancers, 106 genes had physical CRE gene looping and marked transcriptional output that was specific to the LAL-B cells. The selected list included genes that have been previously implicated in B-type ALL and hematopoietic malignancies, such as EBF1, MYB, ETS1, MYC, IRF2, IRF4 [16], and deoxycytidine monophosphate deaminase (DCTD) (Fig. 5B). Strikingly, the expression outcomes of the selected genes were evaluated using two independent datasets covering a cumulative number of 1474 patients [27, 28] and showed marked transcriptional output in all patients regardless of disease subtype (Fig. 4C, Fig. S6C). Our findings suggest that the identified enhancers play a pivotal role in maintaining a broad spectrum of oncogenes in BCP-ALL.

Next, we measured the essentiality score [29] of each upregulated gene by screening the entire set of cell lines (*N* =  ~ 1000 cell lines), which included 11 B-ALL cell lines (Fig. S6D). We selected the top 100 most dependent cell lines for each given gene and counted the number of ALL-B cell lines included in the selection to identify the genes affecting BCP-ALL fitness more than the others (Fig. 5D). Finally, we observed that two genes exhibited marked specificity for ALL-B cell line viability together with a single enhancer-promoter interaction: DCTD and MYB (Fig. 5D-E).

Together, these data demonstrate the ability of our approach to capture qualitative properties of BCP-ALL evolution by identifying previously unknown enhancers that produce eRNA and engage in physical interactions with 106 target genes essential for the BCP-ALL phenotype.

### MYB de novo activated enhancers are important regulatory elements in BCP-ALL

The transcription factor MYB plays a key role in the regulation of hematopoiesis [30, 31]. Single-cell data showed that MYB is highly expressed in myeloid development and epithelial cells, whereas it is transcriptionally silent in most lymphoid cells (Fig. S7A). In addition, several solid tumors show aberrant MYB expression [32] (Fig. S7B). Of note, qRT-PCR analysis of BCP-ALL patients revealed increased gene expression in the onset and relapse groups compared to healthy and remission groups (Fig. S7C).

**Fig. 6:**
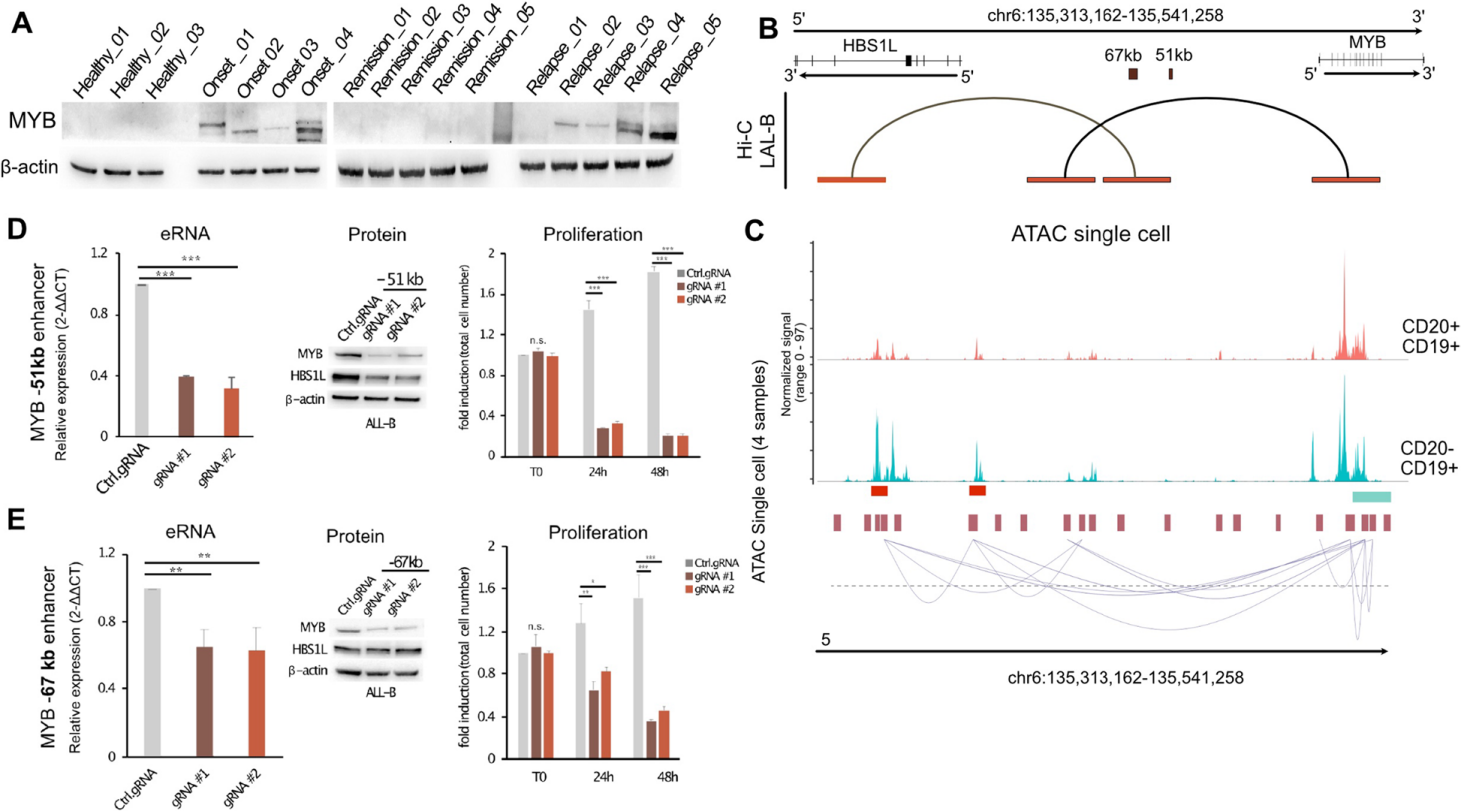
Myb enhancers sustain BCP-ALL progression. **A** Relative expression of Myb protein determined by Western Blot (WB) in healthy (*N* = 3), onset (*N* = 4), remission (*N* = 5), and relapse (*N* = 5). β-actin was used as loading control. **B** Chromatin looping identified by Hi-C Promoter-Capture sequencing at the MYB/HBS1L genomic window. Selected CRE elements are depicted in the dark brown boxes. Red boxes show looping genomic interactors identified by JUICER. **C** The normalized scATAC-seq signal for the region chr6-135,109,357–135186544, MYB enhancers (67kb and 51kb) are highlighted with red bars. Putative loops detected in the CD19 + CD20- with co-accessibility > 0.25 are shown under the profile of the signal in the same genomic region. **D** Left, quantitative RT-PCR (qRT-PCR) analysis for Myb expression performed in B-ALL cells following CRISPR/Cas-9 of -51 kb region using two different gRNAs (#1-#2), compared to a control gRNA. Relative fold changes were determined by the comparative threshold (ΔΔCt) method using β-actin as endogenous normalization control. Data are presented as mean ± SD of three independent experiments: middle, WB with the indicated antibodies in control gRNA and -51 kb gRNA #1 and #2. β-actin was used as loading control; right, cell number analysis of control cells and -51 kb depleted cells at different time points. Data are presented as mean ± SD of three independent experiments. **E** Left, qRT-PCR analysis for Myb expression performed in B-ALL cells following CRISPR/Cas-9 of -67 kb region using two different gRNAs (#1-#2), compared to a control gRNA. Relative fold changes were determined by the comparative threshold (ΔΔCt) method using β-actin as endogenous normalization control. Data are presented as mean ± SD of three independent experiments. middle, WB with the indicated antibodies in control gRNA and -67 kb gRNA #1 and #2. β-actin was used as loading control; right, cell number analysis of control cells and -67 kb depleted cells at different time points. Data are presented as mean ± SD of three independent experiments. **P ≤ 0,01, ***P ≤ 0,001 by Student’s* t*-test

Recent studies have reported that MYB expression is tuned by the activity of a CREs cluster dwelling in the intergenic region spanning 135 kb between MYB and HBS1L genes [33] specifically, enhancers at -88, -84 and -71 and 38 to the MYB promoter are critical regulators of erythropoiesis [34] and leukemic phenotype [35]. Our strategy identified two new enhancer elements within the 135 kb MYB-HBS1L region (enhancer at 67 kb and enhancer at 51 kb to the MYB promoter), significantly more active at onset and relapse of BCP-ALL (Fig. S7.D bottom). Thus, we collected healthy and BCP-ALL samples at the onset, remission, and relapse (*N* = 17 samples) and evaluated MYB protein expression (Fig. 6A). Our data clearly show that the MYB protein significantly emerges in the onset and relapse samples, while dramatically reducing in a healthy-like state after treatment. According to the results of Promoter Capture Hi-C sequencing, the selected elements located at 67 kb and 51 kb distance loop towards the MYB and HBS1L genes, as shown in Fig. 6B. The single-cell ATAC-seq data revealed that CD20- cells were much more enriched in those sites compared to CD20 + cells. Moreover, Cicero [36] predicted the same looping towards the MYB promoter, as identified by the Hi-C Promoter Capture in bulk tissues. Qualitative analysis showed that the 51 kb and 67 kb enhancers were rarely clonal in healthy tissues (Fig. S7C, violin plot). The clonality score significantly increased in the passage from the healthy to the onset state. The enhancer region at 51 kb showed binding of both RUNX2 and ERG TFs while enhancers at 67 kb only RUNX2. Then, inactivating these two distal enhancers using CRISPR/Cas9 significantly reduced eRNA transcription (Fig. 6D left and 6E left). While the inhibition of the-51 kb region negatively reduced both the protein and gene production of MYB and HBS1L, the inactivation of the -67 kb region inhibited only the MYB gene (Fig. 6D middle, E middle, and Figs. S7E-F). Importantly, these results were associated with a significant reduction in the growth rate compared to control cells (Fig. 6D right, E right). Collectively, our results identified two previously unknown activated enhancers in BCP-ALL, which significantly modulated MYB expression and tumor cell proliferation, together with BCP-ALL growth and progression.

### A distal element in BCP-ALL regulates DCTD expression

**Fig. 7:**
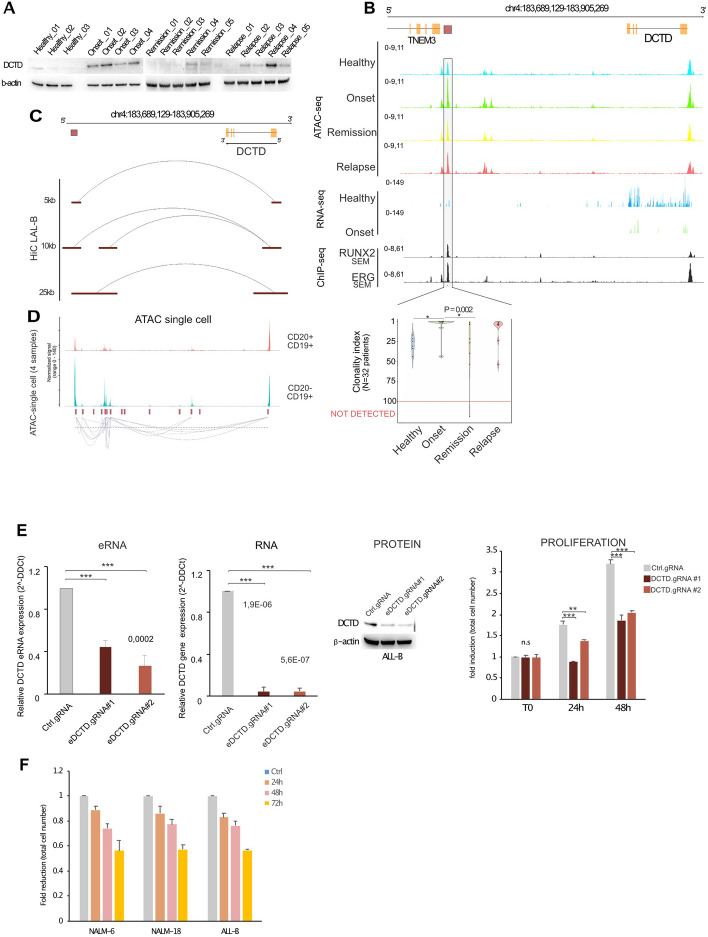
DCTD enhancer is a dominant clone of BCP-ALL progression. **A** Relative expression of DCTD protein determined by Western Blot in Healthy (*N* = 3), Onset (*N* = 4), Remission (*N* = 5), and Relapse (*N* = 5). β-actin was used as loading control. **B** ATAC-seq, RNA-seq profiles of our patient cohort at Healthy Onset, Remission, Relapse (ATAC-seq) and at Healthy and Onset (RNA-seq) at the TNEM3/DCTD genomic window. ChIP-seq of RUNX2 and ERG in SEM cell lines and ChIP-seq of RUNX1 in Kasumi and Karpas cell lines. Black boxes show the identified CREs within the window. Light grey windows highlight selected CREs experimentally validated. Together with violin plots depicting the Clonality index of the given CRE in the patient cohort. Pval represented at the top of each violin plot group is obtained by applying the Kruskal–Wallis chi-squared. The statistical test applied: Pairwise Wilcoxon rank-sum test. * = Pval < 0.05. Color legend of violin plot: Blue = Healthy samples; Green = Samples at Onset; Orange = Samples at Remission; Relapse = Samples at relapse. **C** Chromatin looping identified by Hi-C Promoter-Capture sequencing at the TNEM/DCTD intergenic region (top. Selected CRE element is depicted in the dark brown box. Red boxes show looping genomic interactors identified by JUICER at different resolutions. **D** The normalized scATAC-seq signal for the region chr4:183,689,129–183,905,269. Putative loops detected in the CD19 + CD20- with co-accessibility > 0.25 are shown under the signal profile in the same genomic region. **E** Left, qRT-PCR analysis of DCTD eRNA expression (eDCTD) or DCTD gene expression in BCP-ALL cells following CRISPR/Cas-9 with two different gRNAs (#1- #2) compared to a control gRNA. Relative fold changes were determined by the comparative threshold (ΔΔCt) method using β -actin as endogenous normalization control. Data are presented as mean ± SD of three independent experiments; middle, WB for DCTD in BCP-ALL cells to evaluate CRISPR/Cas-9 efficiency. β–actin was used as loading control; right, cell number analysis was performed in B-ALL cells treated as in D at different time points. Data are presented as mean ± SD of three independent experiments. **P ≤ 0,01, ***P ≤ 0,001 by Student’s *t*-test. **F** NALM-6, NALM-18, and LAL-B cells were treated with 10 µM Tetrahydrouridine (THU), harvested at different time points, and analyzed by Countess Automated Cell Counter. Data are presented as mean ± SD of three independent experiments. ***P ≤ 0,001 by Student’s *t*-test

We identified a critical enhancer that regulates DCTD gene expression. DCTD is a key enzyme in the synthesis of genetic material and catalyzes the deamination of dCMP to dUMP, the nucleotide substrate for thymidylate synthase [37]. Because of its fundamental role, DCTD is ubiquitously expressed in all healthy human and neoplastic cells (Figs. S8A, S8B, and S8C). However, its role in cancer is controversial and partially understood (Fig. S8C) [38] and was markedly downregulated in Acute Myeloid Leukemia (Fig. S8C). DCTD KO rarely affected cell proliferation, and only B-lymphoblastic leukemia cells were affected by DCTD depletion [39] (Figs. 6D-E). Data from our cohort showed that the expression of the DCTD gene was strongly increased in patients with BCP-ALL at both the protein and RNA levels (Fig. 7A and S8E), specifically during the onset and relapse stages, respectively. DCTD depletion by siRNA affected the proliferation of primary LAL-B cells (Fig. S8F). ATAC-seq profiling of our patient cohort identified a region of 108 kb from the DCTD promoter (Fig. 7B top), and Promoter capture of Hi-C in LAL-B cells confirmed that this region physically interacted with the DCTD promoter (Fig. 7C-D). Strikingly, this region was found to be much more accessible in patients with leukemia than in healthy controls, suggesting its involvement in increasing DCTD expression in this disease (Fig. 7C). In addition, this region was dynamically modulated depending on the disease stage (Fig. 7B and S8G). Notably, CRISPR/Cas9 KO of the selected regulatory region showed significant downregulation of eRNA and DCTD RNA production, followed by protein clearance and lower cell proliferation capacity (Fig. 7E). We then monitored the cell viability of 3 different BCP-ALL cell lines upon the usage of an anti-DCTD drug tetrahydrouridine (NCI Thesaurus Code: C868) at 10 µM showing a strong downregulation in the cell viability (Fig. 7F). Altogether, these data prove that a productive enhancer drives DCTD expression and protein translation placed 108 Kb upstream of the DCTD promoter, which is clonally amplified during cancer initiation and recurrence.

## Discussion

While much has been learned about chromatin regulation in cultured cancer cells, epigenomic studies of primary cancer tissues have provided valuable insights into the genuine regulatory specificity of cancers. In this study, we utilized a longitudinal cohort of clinically annotated patients to examine the cis-regulatory elements and explore the function of enhancers in the development and progression of BCP-ALL. Our findings demonstrate that the phenotypic heterogeneity of BCP-ALL is sustained by activating a considerable number of previously unknown enhancers. We discovered over 120,000 active CREs that contribute to the heterogeneity of BCP-ALL and found that cell differentiation occurs independently of chromosome abnormalities. This differentiation leads to the emergence and repression of specific chromatin states in the different stages of BCP-ALL cancer.

**Fig. 8:**
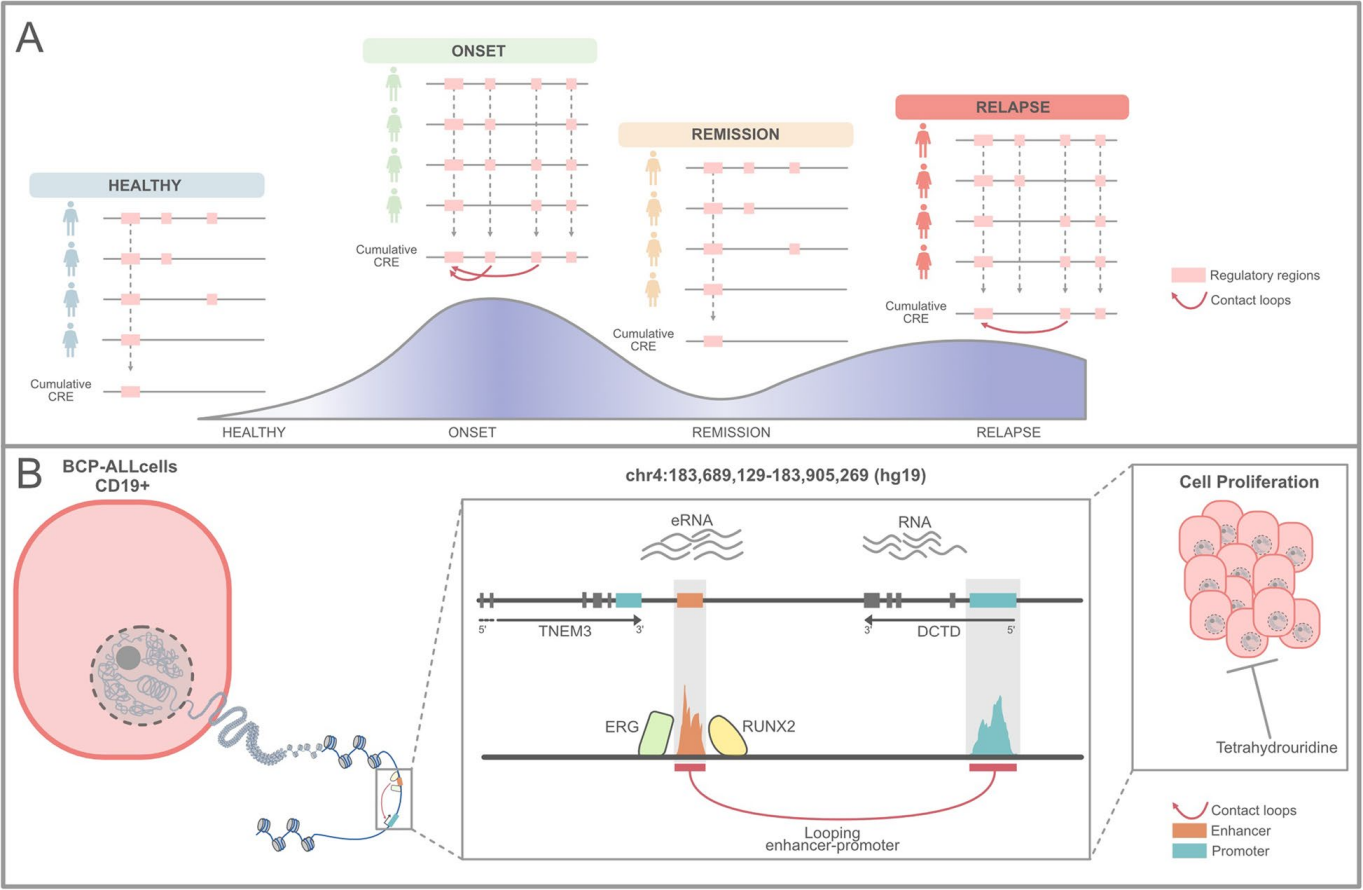
Proposed model. **A** Data from the study indicate that the CRE repertoire undergoes rewiring during BCP-ALL progression. **B** We demonstrate at onset and relapse that the DCTD enhancer strongly transcribes eRNA, which then loops towards the DCTD promoter to enhance RNA transcription. The DCTD enhancer is silent at remission state. DCTD protein sustains cell proliferation, but its inhibition is possible with tetrahydrouridine, a drug that specifically targets DCTD protein

Our study identified approximately 11,000 stage-specific CREs that support the BCP-ALL phenotype, including 5220 CREs (selected as C1 and C2) that were active only at the onset and relapse but silent in healthy and remission samples. Through a multi-omics integrative approach, we investigated the role of 108 CREs with enhancer characteristics and analyzed their long-range gene regulatory interactions with their target genes. Among the 106 identified target genes, some have been previously implicated in hematopoietic malignancies, such as EBF1, MYB, and ETS1, while others, including DCTD, have not been previously linked to BCP-ALL. Notably, most of the enhancer-gene relationships we identified have not been previously linked to lymphoid malignancy, yet our results demonstrate that these cognate target genes are essential in all BCP-ALL cases, as shown with the MYB and DCTD genes. We experimentally demonstrated that MYB and DCTD enhancers are dynamically regulated in relation to the disease stage and play a key role in determining the transcriptional and translational output of the target genes (Fig. 8).

The impact of CRE engagement on cancer development and progression is still not well understood, likely due to the lack of methods that systematically assign a function to each CRE and define its role in cellular regulatory networks. In this study, we addressed this issue by generating data that provided a comprehensive characterization of the landscape and functions of CREs across different stages of BCP-ALL. We constructed the most up-to-date accessibility map of BCP-ALL in relation to publicly available transcriptomic and HiC data from a unique primary cell line model. Our results demonstrated that enhancer engagement and eRNA production are critical regulatory mechanisms in disease progression.

A major limitation of our approach is CREs selection, which relies on the integration of eRNA data to identify the productive/activated enhancers, which produced a clear reduction of probed CREs (from 5220 to 108 CREs). Although eRNAs are increasingly recognized to play important roles in regulating transcriptional gene circuitry in human cancers, their detection is limited by standard sequencing approaches. To systematically detect eRNA, it is necessary to sequence at ~ 200 M reads per sample because of their poor viability. The generation of a high depth sequenced transcriptome dataset would strongly benefit research in this field and support the notion that eRNAs per se may serve as useful and highly precise therapeutic targets for cancer intervention. eRNA expression is highly specific across tissues [40] and cancer types [14, 41]. Targeting eRNAs may confer a superior advantage over gene/protein targeting because their inhibition will not affect other irrelevant tissues and, more importantly, does not completely abrogate the expression of essential target genes. Editing of the newly identified eRNA sequence controlling the Myb-HBSL1 complex and DCTD expression univocally resulted in the inhibition of blast cell viability and proliferation.

Further functional studies of the individual CREs described in this study will be crucial for a better understanding of their role in the development and progression of BCP-ALL. In addition, expanding the patient dataset will be necessary to categorize the activity of CREs more firmly in different disease subtypes. A key future goal will be to characterize the dynamic behaviors of CREs at the single-cell level in longitudinal matching tissues to determine whether they exhibit plastic behaviors in response to treatment or represent the surviving cell subpopulation.

## Conclusion

The findings of this study provide compelling evidence for the value of an epigenetic-based approach in identifying novel tumorigenic elements that can be targeted based on cancer phenotype. By characterizing regulatory elements that are selectively accessible and functionally significant only in the relapsed phenotype, we have the potential to develop new therapeutic strategies that can complement existing treatments. This approach, which prioritizes targeting the key mediators of cancer cell states rather than specific genotypes, represents a promising avenue for the development of effective therapeutic interventions. Overall, these results have the potential to transform cancer treatment by adding focus toward the epigenetic alterations that drive the disease rather than solely relying on genetic mutations and transcriptome analysis.

### Supplementary Information

Supplementary Material 1.

Supplementary Material 2.

Supplementary Material 3: Table 2. Enhancer target genes looping.

Supplementary Material 4.

## Data Availability

Raw sequencing data are available on the GEO portal at GSE214916. Raw data from western blots and experimental essays are available at https://gbox.garr.it/garrbox/index.php/s/5isuYyvnqeq6QqY.

## Abbreviations

BCP-ALL: B-Cell Precursor Acute Lymphoblastic Leukemia
eRNA: Enhancer RNA
BM: Bone Marrow
TSS: Transcription Starting Site
CRE: Cis-Regulatory Element
FDR: False Discovery Rate
RI: Ranking index
PI: Penetrance Index
DCTD: Deoxycytidine monophosphate deaminase
TF: Transcription Factor
