# Supplementary material for: Enhancer engagement sustains oncogenic transformation and progression of B-cell precursor acute lymphoblastic leukemia
Source: J Exp Clin Cancer Res. 2024 Jun 27;43:179. doi: 10.1186/s13046-024-03075-y (PMC11210131; doi:10.1186/s13046-024-03075-y)
Supplement: Supplementary file 1 — Supplementary Material 1. [file 13046_2024_3075_MOESM1_ESM.pdf]

SUPPLEMENTAL METHODS

CRISPR-Cas9 **experiments**

Lentiviral supernatants were generated into HEK293T cells by transient co-transfections of lentiviral CAS9-RFP Lenti Plasmid (Merck, USA) construct and appropriate amount of packaging vectors (Mission Lentiviral Packaging Mix, Sigma-Aldrich, USA) by TRANS-IT (TRANS-IT X2, Dynamic delivery System, Mirus, USA) following the manufacture's protocol. After 48 hours supernatants were collected and employed to LAL-B cells in Retronectin (Takara Bionic Otsu, Shiga 520-2193, Japan) pre-coated no tissue culture 24-well plates (Falcon, BD, USA). Viral particles were centrifuged at 2000g, 90 minutes, at 32°C. Cells were harvested, washed with PBS (Pan Biotech, DH) and replated in RPMI (EuroClone, IT) with 10% FBS (Gibco, USA). After 48 hours, infected cells were sorted following RFP reporter signal to obtain a pure population. LAL-B/CAS-9 clone was transduced, following the Retronectin protocol, with lentiviral particles carrying single CRISPR guide RNA (Custom CRISPR gRNA Plasmid DNA, Merck, USA) directed to DCTD eRNA, -67 kb eRNA and -51 kb eRNA. CTRL-CRISPR gRNA was used as negative control.

gRNAs sequences:

-67 kb cMyb

5'-AAGAGGAAAAGGCGAGAAT-3'

5'-AGAGGAAAAGGCGAGAATC-3'

-51 kb cMyb

5'-TCATTGCTATATGTAGGTA-3'

5'-GCAAACGAAACACGACTCC-3'

DCTD

5'-ATACTCACGCCCGAGAGTC-3'

5'-AGGCTGCATCATCTTCAAA-3'

CTRL

5'- CGCGATAGCGCGAATATATATT-3'

siRNA

siRNA experiments of DCTD expression were performed by transfecting a specific pool of three double-stranded RNA oligonucleotides (siDCTD, cat. n. 1299003 – HSS102676, HSS1026777, HSS102678) or a control sequence (siControl, cat. n. 12935300) (Stealth, Thermo Fisher Scientific) using Amaxa 4D-Nucleofector X kit L (Lonza).

An antisense oligonucleotide targeting DCTD eRNA (CACGGAGCAUGGCAACCUGCAAACA) was purchased from Eurofins Genomics.

Total cellular extracts and western blotting

Total cellular extracts were obtained as described in Bruno et al.[1] Proteins (25 μg) were separated by electrophoresis and transferred onto nitrocellulose membranes. After a blocking step in 5% nonfat-dried milk in 0.1% Tween-PBS, membranes were incubated with primary antibodies overnight at 4°C. After three washes in 0.1% Tween-PBS, membranes were incubated with the appropriate HRP-linked secondary antibodies (Bio-Rad) at room temperature for 45 min, washed with 0.1% Tween-PBS and analyzed by chemi-luminescence (GE Healthcare Life Science). Images were acquired and quantified using Alliance Mini HD6 system by UVITEC Ltd, Cambridge, equipped with UVI1D Software (UVITEC, 14-630275). The rabbit polyclonal antibodies used were: c-Myb (D2R4Y, Cell Signaling), DCTD (ab183607, Abcam), HBS1L (Proteintech). Mouse monoclonal antibody was b-actin (clone AC-15, Sigma-Aldrich).

RNA isolation and quantitative real-time PCR

Total RNA was isolated from cells using EuroGOLD TriFast reagent (Euroclone) according to the manufacturer's instructions. cDNA was synthesized from equal amount of RNA by reverse transcription using M-MLV reverse transcriptase (Thermo Fisher Scientific) and a mixture of

random primers (Thermo Fisher Scientific). This single-stranded cDNA was then used to perform quantitative real-time PCR (qRT-PCR) with specific primers using PowerUP SYBR Green 2x Master Mix (Thermo Fisher Scientific) on a 7500 Fast Real-Time PCR System (Applied Biosystems), following the manufacturer's instructions. Data were processed using the 7500 software v2.0.6 (Applied Biosystems). Relative fold changes were determined by the comparative threshold (DDCt) method using b-actin as endogenous normalization control[2]. Data are presented as mean ± SD of three independent experiments, performed in duplicate. Specific primers employed in qRT-PCR amplifications are listed in Table X. 11846609

ATAC-seq analysis and differential enrichment analysis

The ATAC-sequencing reads quality was assessed with FastQC v0.11.9 (http://www.bioinformatics.babraham.ac.uk/projects/fastqc/). Reads were aligned to the reference genome hg19 using bowtie[3] v2.3.5.1 with default parameters. The conversion from sam to bam file was performed through view function of SAMtools[4] v1.7. BAM files were deduplicated with GATK v4.1.9.0 markDuplicates with default parameters. ATAC-seq peaks were called by MACS2 v2.2.6 with parameters *--format AUTO --nomodel --shift -100 --extsize 200 -B --SPMR --call-summit -q 0.01 -g hs*. BigWig (bw) files were obtained from the BedGraph (bdg) files with bedGraphToBigWig[5] v4 with default parameters. Finally, all peaks matching blacklisted regions (downloaded from https://www.encodeproject.org/files/ENCFF001TDO/@@download/ENCFF001TDO.bed.gz) were removed with the function *intersect* of bedtools[6] suite v2.29.2.

Multidimensional scaling

Multidimensional scaling (MDS) was carried out on a normalized table in which each row is a peak of the master list, and each column represents a different sample. The reads count was performed with bedtools *multicov* v2.29.2.

The reads count per peak was normalized through the R package *edgeR*[7] v3.36.0 and the obtained TMM was transformed through the log2(TMM+1) formula.

Finally, MDS was performed with the *cmdscale* function of *stats* R package v 4.1.2 applied at the samples distance matrix. The distance metric chosen is the following:

$$DistanceMatrix[i,j] = \frac{\sum | A[,i] - \mathrm{B}[,\mathrm{j}]|}{n}$$

Both i and j are samples and n is the total number of peaks.

The scatterplot was performed on first-second and first-third components through ggplot2 R package v 3.3.5.

Differential analysis of accessibility profiling of BCP-ALL

We first built a master list of the accessible regions identified in the profiling of each patient. Narrow peak files of each sample were concatenated, sorted and merged with bedtools *merge* function to finally obtain a list of all accessible region's profiles in at least one patient. We then performed the differential analysis of samples by building a matrix of the peak read count of each sample on the master list. In order to accomplish the reads count the master list and all the samples' bam were used as parameter of *bedtools multicov*. The count normalization was performed with the R package *edgeR* v3.36.0. Two comparisons were performed: Healthy vs Onset and Healthy vs Relapse. Peaks with differences in the means were extracted with *extractTest* edgeR function, and the p-value was corrected through the False Discovery Rate (FDR) method. Finally, the selected peaks whit FDR <= 0.0001 were divided into four groups (-log10(FDR) ≤ 1, -log10(FDR) ≤ 2, -log10(FDR) ≤ 3, -log10(FDR) ≤ 4) and visualized through a scatterplot performed with ggplot2 R package. To provide further significance to the differential analysis, we performed the randomization of the dataset by applying 100 random sample selections. At each iteration, we selected 17 samples randomly selected amongst our cohort of BCP-ALL samples (N=32). At each randomized set, we performed differential analysis as described above by divide the 17 selected sample into two groups: 6 Healthy and

11 Onset. Ontology analysis of the differential subset of peaks was performed by using GREAT[8] v3.0.0. Peaks annotation was performed on hg19 human genome and the basal plus extension was chosen with the following parameters: proximal 5.0 kb, upstream 5.0 kb and plus Distal up to 100.0 kb).

Clonality and Penetrance index scoring strategy and relative analysis

The relationship between RI and SI was displayed through a boxplot obtained with the seaborn python library v0.11.2. Samples were divided into the relative disease group (Healthy, Onset, Remission, and Relapse), and the RI median value was calculated for each peak considering only peaks with RI different from 0.

The linear regression for each status (Healthy, Onset, Remission, and Relapse), the median Clonality index, and the Penetrance index data were fitted with the *sklearn LinearRegressor* model. Finally, the R^2 was calculated on the fitted model. Moreover, a Linear Regressor and R^2 were calculated, gathering all the data of each status. Both models and R^2 were computed through an in-house python script relying on the Scikit-Learn python library v 1.0.2.

To assess the composition of cancer stages in each SI, we divided peaks from the master list into 32 groups (from SI=1 to SI=32). In each group, we calculate the percentage of peaks belonging to every disease group (Healthy, Onset, Remission, and Relapse). The stacked bar plot was used to display the results.

To assess the genomic distance from detected peaks and relative nearest TSS we divided the peaks into four lists, one for each disease group, containing only peaks detected in at least one sample within each group. We ran each list to the *annotatePeaks.pl* function included into HOMER[9] suite. Then, peaks were divided into five groups of distance relative to the closest TSS (<5kb , 5kb-20kb, 20kb-100kb, >100kb). Finally, for each group was calculated the percentage contribution at each distance group and plot with an in-house R scripts. The HOMER results described above were also used to classify peaks according to the genomic

context. Hence, peaks were divided into six classes (Non-Coding, Promoter, Exon, Intron, TTS, and UTR) according to the HOMER output.

Observed and Expected (O/E) relationship of peaks at disease stage

We measured the significance of the observed peaks in our cohort and their relationship with the disease stage. A count of the observed number of peaks belonging to each Penetrance score was performed. The expected number of peaks for each status per SI was calculated applying this formula:

$$Expected\ number\ of\ peaks\ =\ \frac{Tr\ \cdot\ Tc}{Tr\ +\ Tc}$$

With:

Tr: total number of peaks in a row

Tc: total number of peaks in a column

Finally, we performed the observed-expected ratio for each disease group per SI.

Assessment of peak dynamics during BCP-ALL evolution

Peaks were stratified according to their relative impact in driving cancer onset and relapse. We first assigned the median of the Clonality index and the Penetrance index to each peak of the master list. The final matrix was then populated with peak coordinates (hg19) at the rows and Clonality index median and penetrance index of each status at the columns (eight columns).

The selection criteria to prioritize the phenotypical peak drivers are the following:

Peaks that harbor Onset Penetrance Index ≥ 9 and Healthy Penetrance Index ≤ 3 were selected. Peaks that show an increase of at least 20 in the difference between the Healthy Clonality index median and Onset Clonality index were selected. Finally, peaks that harbor an increase of at least 20 in the difference between the Remission Clonality index median and Relapse Healthy Clonality index were selected. Finally, we obtained a list of unique 11.077 unique peaks. Enrichment trends of the selected peak at each cancer stage were plotted as a boxplot with an in-house r script.

Density distribution of phenotypically driver peaks

We measured the relationship of the selected peaks according to the cancer stage by calculating the density distribution of the penetrance score. We first calculated the ratio of each peak by the number of total samples at the disease stage and obtained a standardized score ranging between 0 to 1, where 0 is associated with a silent state and 1 to activation in all the patients in the disease stage cohort. Finally, we plotted the density distribution of peaks across the four-cancer stages through the *plot_density* function of the ggplot2 R package v 3.3.5.

Heatmap of peak dynamics during BCP-ALL evolution

A heatmap was performed to assess the read count of the 11.077 selected peaks across all 32 samples. First, a read count of these sites was performed through the bedtools *multicov* v2.29.2 with standard parameters. The obtained reads count was normalized through the R package edgeR v. 3.36.0 in order to obtain the log2(TMM+1) metric. Finally, the TMMs were scaled, and the visualization was performed through an R script through the ComplexHeatmap R package v. 2.10.0. The cluster detection was accomplished according to the Ward2 hierarchical clustering to retrieve four final clusters classified as C1, C2, C3, and C4. The similarity across samples was evaluated through the Euclidean distance.

Transcription Factors Identification

Peaks belonging to the selected four clusters above (C1, C2, C3, C4) were analyzed in order to infer the putative transcription factor able to bind towards these genomic regions. The HOMER findMotifsGenome.pl (parameter: --genome hg19) tool v4.11 was employed. For each of the significant transcription factors identified, we calculated the Observed/Expected ratio and displayed the data as a circular bar plot with an in-house r script.

Integration with TCeA portal data

To infer the number of eRNA productive peaks, we integrated our peak selection with the TCeA[10] portal by downloading the Canonical Enhancer and Super-Enhancer data from

available tumor types. We performed the intersection of the 2 datasets with our peak selection with bedtools intersect tools v2.29.2.

Putative eRNA in the BCP-ALL cohort

In order to assess the amount of actively transcribed enhancers, we performed a bedtools intersect between the TCeA eRNA data and the blacklisted MACS2 output narrowpeak file of each patient. Finally, we plotted the MACS2[11] *signalValue* of the actively transcribed peaks. The violin plot was ordered according to the median of the peaks' enrichment in each patient.

eRNA identification of RNA-seq BCP-ALL cohort

Total RNA sequencing was performed to estimate the eRNA expression across 8 samples (4 Healthy and 4 Onset) at the cancer phenotype's selected regulatory regions. Quality control of the RNA-sequencing reads was obtained through FastQC v0.11.9. Reads were aligned to the reference genome hg19 using STAR[12] (v. 2.7.9a). The conversion from sam to bam file was performed through the view function of SAMtools v1.7.

C1 and C2 clusters (N=5216) were extended with the DHS sites downloaded from https://personal.broadinstitute.org/meuleman/reg2map/HoneyBadger_release/ to quantify the expression of the non-coding genomic regions across the 11 patient RNAseq. We then removed all regions intersecting exonic regions. The read count of the extended non-coding regions (N=4935) was evaluated through the multicov function of bedtools v2.29.2 with standard parameters.

The read count standardization was accomplished through the edgeR v. 3.36.0 in order to obtain the scaled log2(TMM+1) used for the heatmap visualization. The two distinct clusters were carried out by dividing the dendrogram generated from the hierarchical cluster into two clusters. Finally, the sites of the target genes were identified according to the nearest gene inferred by using the HOMER annotatePeaks.pl (parameter: --genome hg19) function v4.11.

Promoter-Capture Analysis

Paired end FASTQ files were aligned against the hg19 genome. The KR-normalized contact matrices and the loop annotation were performed with Juicer[13] v 1.9.9. Loops were called at 5kb, 10kb, and 25kb resolution.

We classified each promoter-CRE looping by intersecting the bedpe file generated by Juicer and the promoter coordinates (downloaded from https://egg2.wustl.edu/roadmap/data/byDataType/dnase/BED_files_prom/regions_prom_E001.bed) using bedtools intersect v2.29.2. We calculated the number of interactions in three different target-anchor loops (promoter-promoter, promoter-NonCoding, and NonCoding-NonCoding). Moreover, we assessed the looping length in genomic coordinates according to the distance from anchor to targets into five classes (<50kb, 50kb-200kb, 200kb-500kb, 500kb-1Mb, and >1Mb).

RNA-seq analysis of LAL-B , B cells during differentiation and public patient datasets

Differential expression analysis across LAL-B cell line and normal B cells[14] (GEO: GSE118165; Table 1). Samples were aligned to hg19 through STAR and quantified with RSEM[15]. Both the alignment and quantification were performed through the nf-core/rnaseq v3.0 from NEXTFLOW v21.05.0-edg[16] with parameters: --aligner star_rsem. The quantification files were normalized through the standard edgeR (v 3.36.0) pipeline. Once the normalization step was carried out, we selected only the target gene of the selected enhancers and compute both the Ward2 hierarchical clustering and the heatmap with the R package ComplexHeatmap v 2.10.0.

In order to assess the gene expression of target genes on a larger cohort, an analysis of transcriptomic profiles of 251 type-B acute lymphoblastic leukemia patients (188 Onset and 63 Relapse) and 1223 patients were performed. Data were downloaded from the TARGET: Acute Lymphoblastic Leukemia (ALL) Phase II (phs000464) and from Li. Et al.[17]. Then the log2 transformation was applied to the fpkm available. Finally, only 149 genes of interest were

retained. The heatmap was performed through the ComplexHeatmap R package. Both columns and rows were clustered, and the Euclidian distance was computed across columns and rows. Then, cluster identification was performed through the Ward D algorithm.

Identification of the enhancer-target genes was manually curated by integrating ENCODE data[18] (ChIAP-pet, H3k27ac ChIP-seq), RNA-seq from TCGA and Promoter-Capture of LAL-B.

ATAC-seq of B cells at multiple differentiation states

B-cell ATAC-seq FASTQ files were available at the GEO GSE118189 (Table 3).

The FASTQ quality was assessed with FastQC v0.11.9. Reads were aligned to the reference genome hg19 using bowtie v2.3.5.1 with default parameters. The conversion from sam to bam file was performed through view function of SAMtools v1.7. BAM files were deduplicated with GATK v4.1.9.0 markDuplicates with default parameters. ATAC-seq peaks were called by MACS2 v2.2.6 with parameters --format AUTO --nomodel --shift -100 --extsize 200 -B --SPMR --call-summit -q 0.01 -g hs. BigWig (bw) files were obtained from the BedGraph (bdg) files with bedGraphToBigWig v4 with default parameters. Finally, all peaks matching blacklisted regions (downloaded from https://www.encodeproject.org/files/ENCFF001TDO/@@download/ENCFF001TDO.bed.gz) were removed with the function intersect of bedtools v2.29.2.

An assessment of read count both for LAL-B cell line, and the normal B-cell was performed through the bedtools multicov function v2.29.2. Finally, the read count file was standardized according to the library size through the edgeR R package v 3.36.0 and plotted through the ComplexHeatmap R package v 2.10.0. Both rows and columns of the resulting heatmap were clustered through the Ward2 unsupervised hierarchical clustering methods computing the distance through the Euclidean distance.

Assessment of selected CRE clonality during BCP-ALL evolution

The clonality trends of the selected enhancer were assessed by analyzing Clonality Index across the four cancer stages and depicted as violin plots.

For each enhancer, the Clonality indexes were plotted, and statistical tests were performed: Kruskal-Wallis rank-sum test followed by Dunn's Test.

Analysis of Transcription Factors (TF)

We inferred the Transcription factor binding to the selected 111 enhancer, by downloading 32 ChIPseq of Transcription Factors: AP1: 8 samples, ATF3 : 4 samples, EBF1 : 2 samples , ELK4 : 2 samples, ERG:1 sample, ETS1 : 2 samples, ETV4 : 3 samples, FRA2 : 2 samples , RUNX1 : 5 samples ,RUNX2 : 2 samples. Data were available in fastq format from the ChIPAtlas[19] (all the downloaded files annotation are gathered in the table 5). The fastq alignment and ChIP-seq seq peak detection were performed as described in the previous sections. For each transcription factor was performed the intersection between the selected enhancer and the blacklisted files with bedtools intersect v2.29.2. Finally, we ranked the TF by the number of the relative signal matching the selected enhancer.

Pan-cancer analysis of H3K27ac ChIP-seq at selected 130 CREs

We downloaded all the available cell lines and primary cancer cell lines profiled for H3K27ac ChIP-seq available in ENCODE. Peak profiles of each BAM file were obtained as the follows: i) sorted with samtools sort v1.7; ii) the duplicated reads were removed with GATK v4.1.9.0 markDuplicates with default parameters; iii) the resulting file was indexed through samtools index v1.7; v) peaks were called through the MACS2 v2.2. callpeak function (parameters: --format AUTO -B --SPMR --call-summits -q 0.01); vi) BigWig (bw) files were obtained from the BedGraph (bdg) files with bedGraphToBigWig v 4 with default parameters. We assigned the Clonality Index to each significant peak as previously described. Clonality index annotation at the selected 130 CREs were shown as a heatmap where rows were arranged to display the enhancer detected in the highest number of cell lines at the top. Moreover, Ward2 hierarchical

clustering was performed on the column and the sample distance was computed with the Euclidean distance.

Gene dependency analysis

The fitness scores (CHRONOS) describing the effect caused by CRISPR knockout of 17.393 genes were downloaded from DepMap Public 21Q3 portal, table name CRISPR_gene_effect.csv. The data were restricted to only genes that resulted up-regulated from the differential analysis between LALB and B cells (N=106) and then plotted CHRONOS score of all available cell lines. The procedure to determine this list of genes was previously described. We further selected the genes exhibiting strong dependency specific to only BCP-ALL cell lines by ranking each CHRONOS score x disease cell lines. Only the genes showing lower CHRONOS score (high dependency) to BCP-LL cell lines (697, JM1, SEM, RCHACV, NALM6, REH, ROS50, SEMK2, HB1119, NALM16, P30OHK) were retained and plotted with an in-house R script.

Multi-omics single-cell analysis

Sequenced data were processed with the Cell Ranger ARC software v. 2.0.2 (10x Genomics). Multiomics reads were aligned to the GRCh38 (hg38) reference genome and quantified through cellranger arc count v. 2.0.2 (10x Genomics). To retain high-quality sequenced cells, we filtered cells according to the following parameters: percentage of reads in peaks > 50; 0.2< TSS enrichment < 20; 0.2<nucleosome signal<2; RNA feature < 10000; RNA reads < 100000; and reads aligning with mitochondrial genome < 20%. RNA seq counts were normalized using scTransform[20] considering the best 5000 features. Identification of source of variation, clustering and enrichment analysis was performed with seurat v5.0.1 e signac v.1.13.0.

ATAC seq counts were normalized through the TF-IDF method, considering all the peaks with at least 5 total counts. Normalized features were then employed for the dimensionality reduction PCA and LSI, respectively, for RNA and ATAC. Samples were then integrated using

Harmony[21] R package (v.1.1.0) according to patient and cancer status with lambda equal to 1 for both. The integrated dimensions were then used to build the shared nearest neighbor graph considering the first 50 RNAseq components and from the 2nd to the 40th ATAC components. The first ATAC seq components were filtered out due to the high correlation with the sequencing depth. Communities were mined using the Leiden algorithm with a resolution of 0.8. Cells were visualized in two-dimensional space according to the Uniform Manifold Approximation and Projection (UMAP) dimensional reduction.

The resulting communities were annotated through the singleR R package (v. 2.4.0) considering the Human Primary Cell Atlas reference of celldex Rpackage (v.1.12.0). To discriminate between healthy and malignant cells we evaluated the SCT normalized expression of MS4A1 marker gene. Cells in the active proliferative state were removed from the analysis to retain only cells in the G1 phase. The cell cycle evaluation was performed through the CellCycleScoring function.

To evaluate the chromatin accessibility score of C1,C2,C3, and C4, we first converted the genomic coordinates from hg19 to hg38 using the LiftOver web tool (https://genome.ucsc.edu/cgi-bin/hgLiftOver), then we used the AddChromatinModule Seurat function with standard parameters. The Seurat object was then converted into a cicero[22] CDS to find loops in the sc-ATAC experiment. The conversion was done with the SeuratWrappers R package (v0.3.2), and the cells were grouped with the make_cicero_cds function with k = 100 and UMAP coordinates.
ATAC seq signals were plotted with the CoveragePlot function. Loops were detected with the run_cicero function and plotted with the plot_connections function, filtering loops with a correlation lower than 0.25.

SUPPLEMENTARY FIGURES

Supplementary Figure 1

A) Stacked bar chart depicting the percentage of ATAC-seq peaks x disease stage (Healthy-Onset- Remission-Relapse) in the function of the genomic distance to the closest TSS. Color legend: Blue= Healthy samples; Green= Samples at Onset; Orange= Samples at Remission; Relapse= Samples at relapse. **B)** Stacked bar chart showing the absolute number of ATAC-seq peaks at each given genomic annotation in Healthy, Onset, Remission, and Relapse groups. Color gradient from Purple to Green: Non-coding, Promoter, Exon, Intron, TTS, UTR. Annotation generated with HOMER suite. **C)** Upset plot of detected peaks at TSS (Promoter-like) proximity among the different groups of patients. X-axis: Intersection combination; Y-axis: the absolute number of detected sites. Color legend: Blue= Healthy samples; Green= Samples at Onset; Orange= Samples at Remission; Relapse= Samples at Relapse; Violet: Barchart of the number of detected sites at each intersection. **D**) Upset plot of differential peaks among all the differential analyses performed. X-axis: Intersection combination; Y-axis: the absolute number of detected sites. Color legend: Blue= Healthy samples; Green= Samples at Onset; Orange= Samples at Remission; Relapse= Samples at relapse. **E**) Ontologies of the differential accessible sites at Healthy vs. Onset (left) and Healthy vs. Relapse right. Top: Biological Process; Middle: Disease Ontology; Bottom: Mouse Phenotype. The analysis is performed with the GREAT tool. Color scheme: Green= Upregulation at Onset; Red= upregulation at Remission **F)** Peak signal normalized across the full cohort of patients at selected genomic windows. Color legend: Blue= Healthy samples; Green= Samples at Onset;

Orange= Samples at Remission; Relapse= Samples at relapse. Clonal CREs at the Onset and Relapse were further selected.

Supplementary Figure 2

a) Line plot of the linear regression between clonality index and the penetrance index among the four disease groups. y-axis = Clonality index, x-axis = Penetrance index. The coefficient of determination of each given linear analysis performed is shown on the bottom right. Color legend: Blue= Healthy samples; Green= Samples at Onset; Orange= Samples at Remission; Red= Samples at relapse.

Supplementary Figure 3

A) Barchart shows the number of the selected peak in relationship with the genomic distance to the closest TSS. **B)** Gene ontology analysis of the selected peaks at three different databases: Biological process (blue); Disease ontology (red); Human Phenotype (violet). Significance is depicted on the x-axis (FDR)

C) UMAP colored according to the twelve clusters detected through the Leiden algorithm with a resolution of 0.8.

D) UMAP with cells grouped according to the cellular cycle phase.

E) UMAP with cells grouped according to the selected regulatory clusters.

F) Violin plot shows peak enrichment over background at eRNA loci in each sample identified by integrating the TCeA portal. Samples are sorted from low (left) to high (right) by the median of peak enrichment. **G)** Upset plot shows the common peaks between the master list of onset, relapse, and LAL-B cell lines. Color legend: Green= Samples at Onset ; Red= Samples at relapse; Violet= LAL-B.

Supplementary Figure 4

A) Heatmap shows the clonality score of the selected CREs (N=118) from H3k27ac ChIP-seq available on 159 cell lines (ENCODE data). Data were gathered by unsupervised clustering at the column and supervised at the rows. Column: Cell Lines Rows: CREs are named by the closest gene. Color legend: Blue= lower clonality index, Red= higher clonality index; White= no signal.

Supplementary Figure 5

A) HiC Promoter-capture sequencing interactions at selected loci among the identified enhancers. Each window shows (orange squares from top-to-bottom) 1) enhancers being part of the Final Selection (occurring in our patient cohort, TCeA, LaLB chip-seq), 2) enhancers part of C1, C2,C3, C4 clusters 3) Promoter-Capture HiC interaction of LaLB cell line split by a resolution of 5kb, 10kb, 25kb. Shadow orange lines represent the enhancer identified in the final selection. From top to bottom: BCL2, EBF1, IRF4, CADM1, GTF3C3 genomic loci.

Supplementary Figure 6

A) Example of Promoter-Capture HiC looping in LAL-B (top) together with signals of (from top to bottom) ATAC-seq of LAL-B, CTCF ChiaPet of K562, Pol2 ChiaPet of K562, ATAC-seq from patients at Healthy, Onset, Remission and Relapse. The genomic window covers the coordinates of MYC locus (chr8:127,025,953-130,946,417), reference genome is HG19. Color legend: Blue= Healthy samples; Green= Samples at Onset; Orange= Samples at Remission; Relapse= Samples at relapse **B)** The heatmap depicts the unsupervised clutering of ATAC-seq

data at the C1, C2, C3, C4 selected loci in Naïve-B cell (light grey), Memory B-cell (sky-blue), Bulk Bcell (dark-grey) and LAL-B (violet). Enrichment data are log2(Z-scaled). Color legend: Blue= low enrichment, Red= high enrichment. **C)** Unsupervised clustering of the Transcriptomic profiles of the selected enhancer-targeted genes in 1223 patients. Color scheme: log2(FPKM). Alteration groups are designed according to the molecular subtypes available in Li et al. (PNAS, 2018). **D)** Dot plot showing the Chronos score (x-axis) of the selected genes (N=106 genes) in all the available cell lines (grey dots). ALL-B cell lines: 697, JM1, SEM, RCHACV, NALM6, REH, ROS50, SEMK2, HB1119, NALM16, P30OHK are colored violet.

Supplementary Figure 7

A) Bar Chart shows the cumulative enrichment of gene expression of single-cell types of the most expressing MYB gene. Colors depict cell groups as follows: Red Blood and immune cell types; Light blue Undifferentiated cells; Blue Glandular epithelial cells; Purple endothelial cells; Orange Pigment cells. Data obtained from Human Protein Atlas portal https://www.proteinatlas.org/. **B)** Box plot shows RNA expression of MYB in cancer tissues from TCGA. Data obtained from Human Protein Atlas portal https://www.proteinatlas.org/.

C) ATAC-seq, RNA-seq profiles of our patient cohort at Healthy Onset, Remission, Relapse (ATAC-seq) and at Healthy and Onset (RNA-seq) at the MYB/HBS1L genomic window, ATAC-seq of LAL-B and H3K27ac ChIP-seq of LAL-B, ChIP-seq of RUNX2 and ERG in SEM cell lines. Black boxes (Top) show the identified CREs within the window. Light grey windows highlight selected CREs experimentally validated. Together with violin plots depicting the Clonality index of the given CRE in the patient cohort. Pval represented at the top of each violin plot group is obtained by applying the Kruskal-Wallis chi-squared The statistical test applied:

Pairwise Wilcoxon rank-sum test. *= Pval< 0.05. Color legend of violin plot: Blue= Healthy samples; Green= Samples at Onset; Orange= Samples at Remission; Relapse= Samples at relapse.

D) MYB mRNA levels were analyzed by qRT-PCR in bone marrows samples of healthy donors (4), or from B-ALL patients at the time of onset (3), remission (3) or relapse (3). Relative fold changes were determined by the comparative threshold method (ΔΔCt) using β-actin as endogenous normalization control. Data are presented as mean ± SD of three independent experiments. ***P ≤0.001. **E)** qRT-PCR analysis for Myb (left) or HBS1L (right) expression performed in B-ALL cells following CRISPR/Cas-9 of -51 kb region or -67 kb region using in each two different gRNAs (#1- #2), compared to a control gRNA. Values were normalized with β-actin mRNA levels using ΔΔCt method. Data are presented as mean ± SD of three independent experiments. ***P ≤0.001, **P ≤0.01 by Student's *t*-test.

Supplementary Figure 8

A) Bar Chart shows the cumulative enrichment of single cell gene expression of different cell types of the most expressing DCTD gene. Colors depicts cell groups are showed on the right legend. Data obtained from Human Protein Atlas portal https://www.proteinatlas.org/. **B)** Protein expression of DCTD. Percentage of patients (y-axis) with high and medium DCTD protein level in different cancer cell types. Color code of the barchart is according to the type of normal organ the cancer originates. Data obtained from Human Protein Atlas portal https://www.proteinatlas.org/. **C)** Gene expression of DCTD gene (y-axis)in cancer (red) vs normal (green) tissues in cancer types available on TCGA portal. Cancer types exhibiting

significance difference are colored red on the cancer type labeL (top). Data obtained from GEPIA2 (http://gepia2.cancer-pku.cn/).

D) Gene expression levels (TPM) of DCTD gene (y-axis) in cancer cell lines grouped by tissue of origin (colors).

E) DCTD mRNA levels were analyzed by qRT-PCR in bone marrow samples of healthy donors (4) or from B-ALL patients at the time of onset (3), remission (3), or relapse (3). Values were normalized with b-actin mRNA levels using ΔΔCt method. Data are presented as mean ± SD of three independent experiments. ***P ≤0.001, **P ≤0.01 **F)** Left**,** WB analysis of total cellular extracts from B-ALL cells transfected with siRNA oligonucleotides targeting DCTD (siDCTD) or a control sequence (siControl). b-actin was used as a loading control. The same B-ALL cells were analyzed for cell number (middle) and for DCTD mRNA levels by qRT-PCR (right). Data are presented as mean ± SD of three independent experiments. ***P ≤0.001, **P ≤0.01. **G)** Boxplots show the Ratio between peaks (N-score) at the selected enhancer and the MYB promoter signal in the patient cohort. Each dot represents a patient. Color legend: Blue= Healthy samples; Green= Samples at Onset; Orange= Samples at Remission; Relapse= Samples at relapse. Statistical tests performed: Kruskal-Wallis rank-sum test followed by Dunn's Test. *P≤ 0.05.

SUPPLEMENTAL REFERENCES

1. Bruno, T. *et al.* Che-1 phosphorylation by ATM/ATR and Chk2 kinases activates p53 transcription and the G2/M checkpoint. *Cancer Cell* **10**, 473–486 (2006).

2. Livak, K. J. & Schmittgen, T. D. Analysis of Relative Gene Expression Data Using Real-Time Quantitative PCR and the 2−ΔΔC T Method. *Methods* **25**, 402–408 (2001).

3. Langmead, B. & Salzberg, S. L. Fast gapped-read alignment with Bowtie 2. *Nat. Methods* **9**, 357–359 (2012).

4. Langmead, B. Aligning short sequencing reads with Bowtie. *Current protocols in bioinformatics* **Chapter 11**, Unit 11.7 (2010).

5. Kent, W. J., Zweig, A. S., Barber, G., Hinrichs, A. S. & Karolchik, D. BigWig and BigBed: enabling browsing of large distributed datasets. *Bioinformatics* **26**, 2204–2207 (2010).

6. Quinlan, A. R. & Hall, I. M. BEDTools: a flexible suite of utilities for comparing genomic features. *Bioinformatics (Oxford, England)* **26**, 841–2 (2010).

7. Robinson, M. D., McCarthy, D. J. & Smyth, G. K. edgeR: a Bioconductor package for differential expression analysis of digital gene expression data. *Bioinformatics* **26**, 139–140 (2010).

8. McLean, C. Y. *et al.* GREAT improves functional interpretation of cis-regulatory regions. *Nature Biotechnology* **28**, 495–501 (2010).

9. Heinz, S. *et al.* Simple combinations of lineage-determining transcription factors prime cis-regulatory elements required for macrophage and B cell identities. *Molecular cell* **38**, 576–89 (2010).

10. Chen, H. & Liang, H. A High-Resolution Map of Human Enhancer RNA Loci Characterizes Super-enhancer Activities in Cancer. *Cancer Cell* **38**, 701-715.e5 (2020).

11. Zhang, Y. *et al.* Model-based analysis of ChIP-Seq (MACS). *Genome biology* **9**, R137 (2008).

12. Dobin, A. *et al.* STAR: ultrafast universal RNA-seq aligner. *Bioinformatics* **29**, 15–21 (2013).

13. Durand, N. C. *et al.* Juicer Provides a One-Click System for Analyzing Loop-Resolution Hi-C Experiments. *Cell Syst* **3**, 95–98 (2016).

14. Calderon, D. *et al.* Landscape of stimulation-responsive chromatin across diverse human immune cells. *Nat Genet* **51**, 1494–1505 (2019).

15. Li, B. & Dewey, C. N. RSEM: accurate transcript quantification from RNA-Seq data with or without a reference genome. *BMC Bioinform.* **12**, 323 (2011).

16. Tommaso, P. D. *et al.* Nextflow enables reproducible computational workflows. *Nat. Biotechnol.* **35**, 316–319 (2017).

17. Li, J.-F. *et al.* Transcriptional landscape of B cell precursor acute lymphoblastic leukemia based on an international study of 1,223 cases. *Proc National Acad Sci* **115**, 201814397 (2018).

18. Davis, C. A. *et al.* The Encyclopedia of DNA elements (ENCODE): data portal update. *Nucleic Acids Res.* **46**, gkx1081- (2017).

19. Oki, S. *et al.* ChIP-Atlas: a data-mining suite powered by full integration of public ChIP-seq data. *EMBO Rep.* **19**, e46255 (2018).

20. Hafemeister, C. & Satija, R. Normalization and variance stabilization of single-cell RNA-seq data using regularized negative binomial regression. *Genome Biol.* **20**, 296 (2019).

21. Korsunsky, I. *et al.* Fast, sensitive and accurate integration of single-cell data with Harmony. *Nat. Methods* **16**, 1289–1296 (2019).

22. Pliner, H. A. *et al.* Cicero Predicts cis-Regulatory DNA Interactions from Single-Cell Chromatin Accessibility Data. *Mol. Cell* **71**, 858-871.e8 (2018).

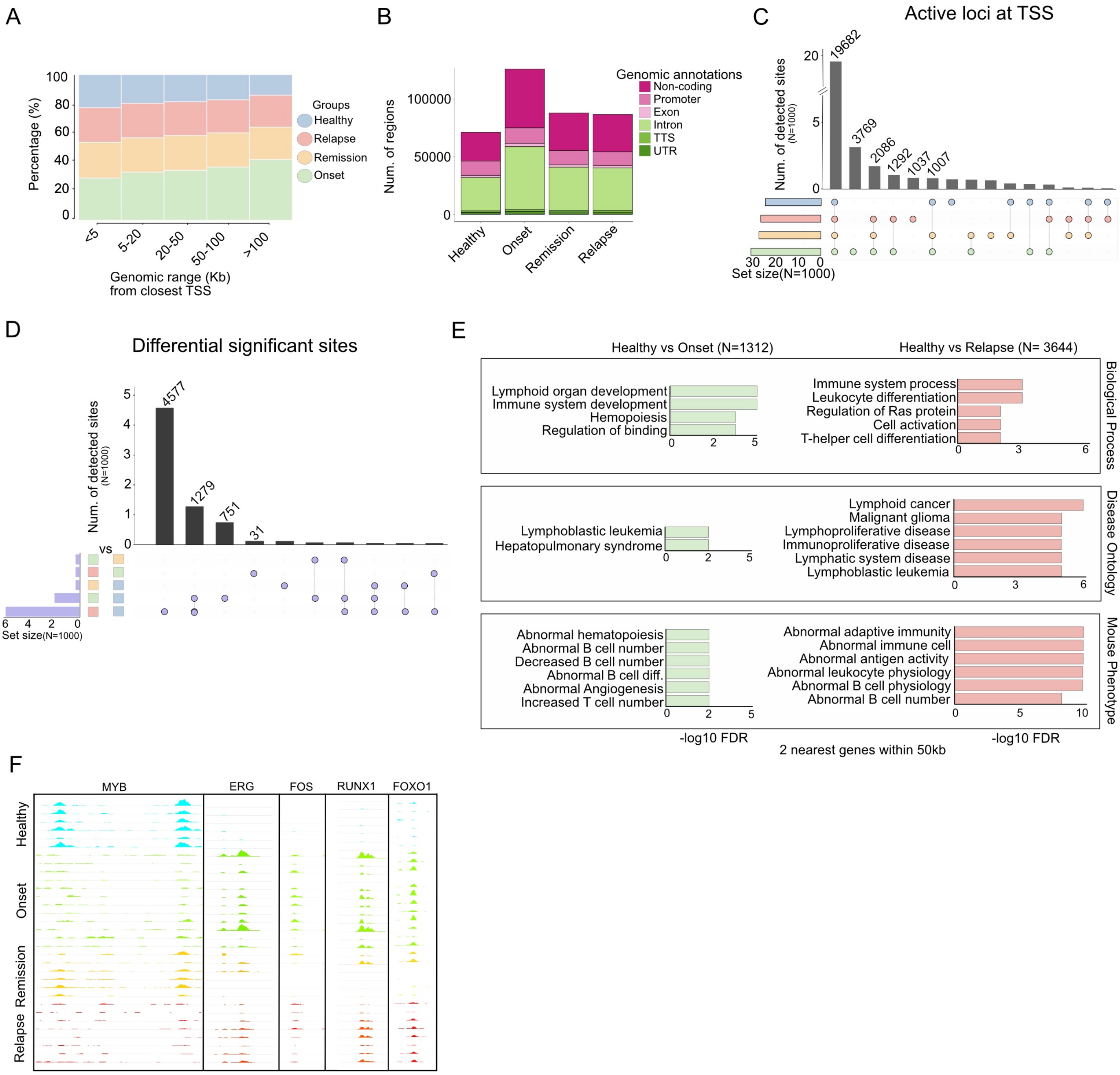

Supplementary Figures 1

A

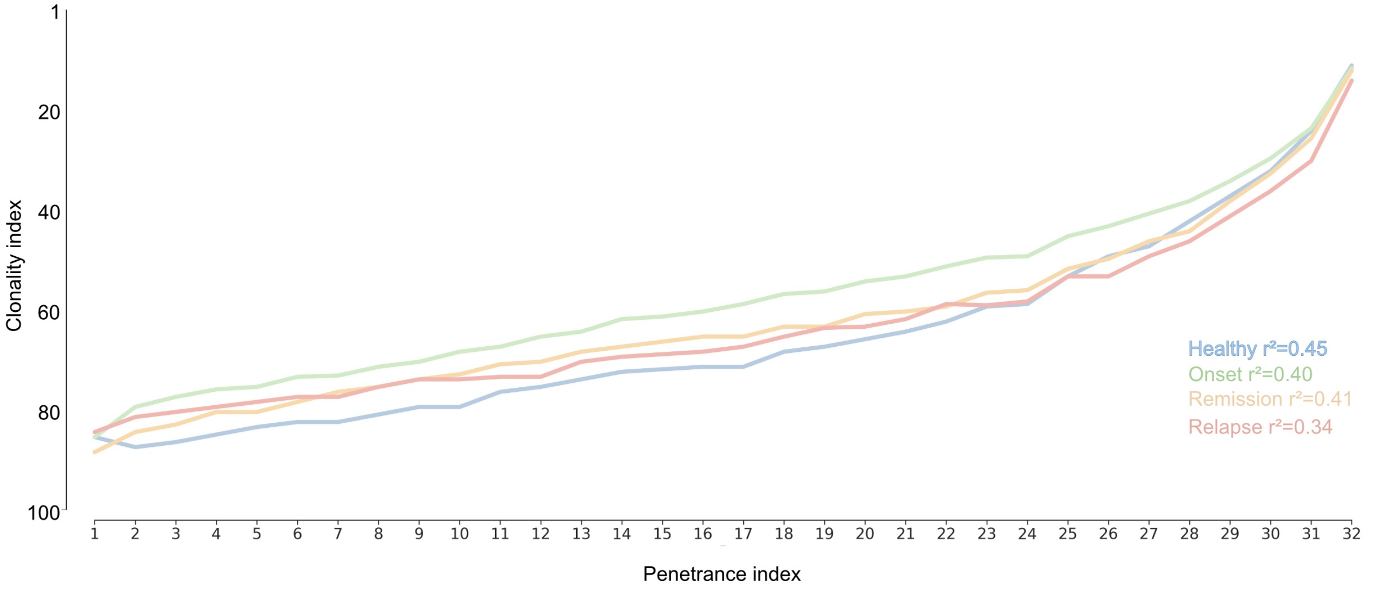

Clonality index
1
20
40
60
80
100
Penetrance index
1 2 3 4 5 6 7 8 9 10 11 12 13 14 15 16 17 18 19 20 21 22 23 24 25 26 27 28 29 30 31 32
Healthy r²=0.45
Onset r²=0.40
Remission r²=0.41
Relapse r²=0.34

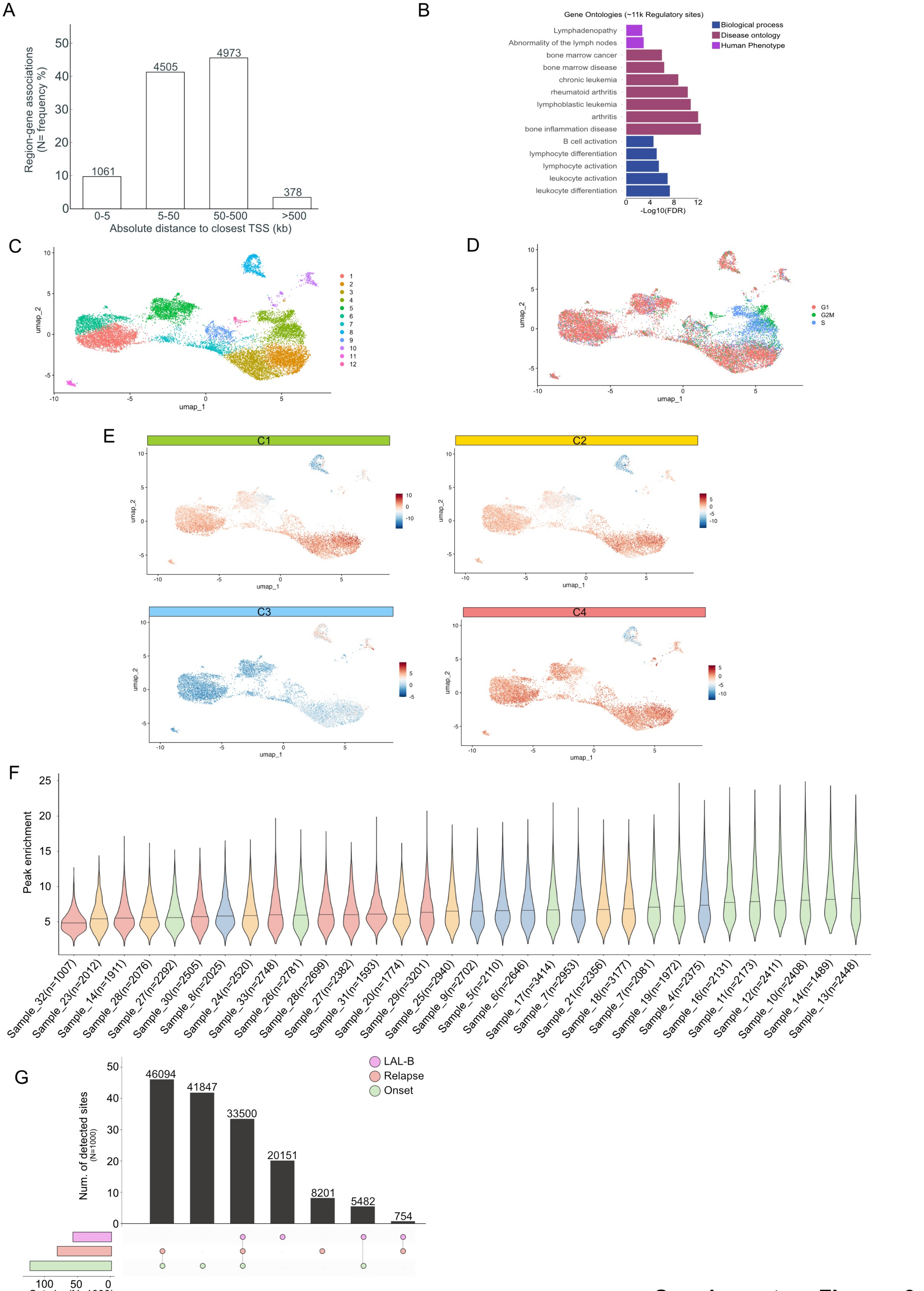

Supplementary Figures 3

A

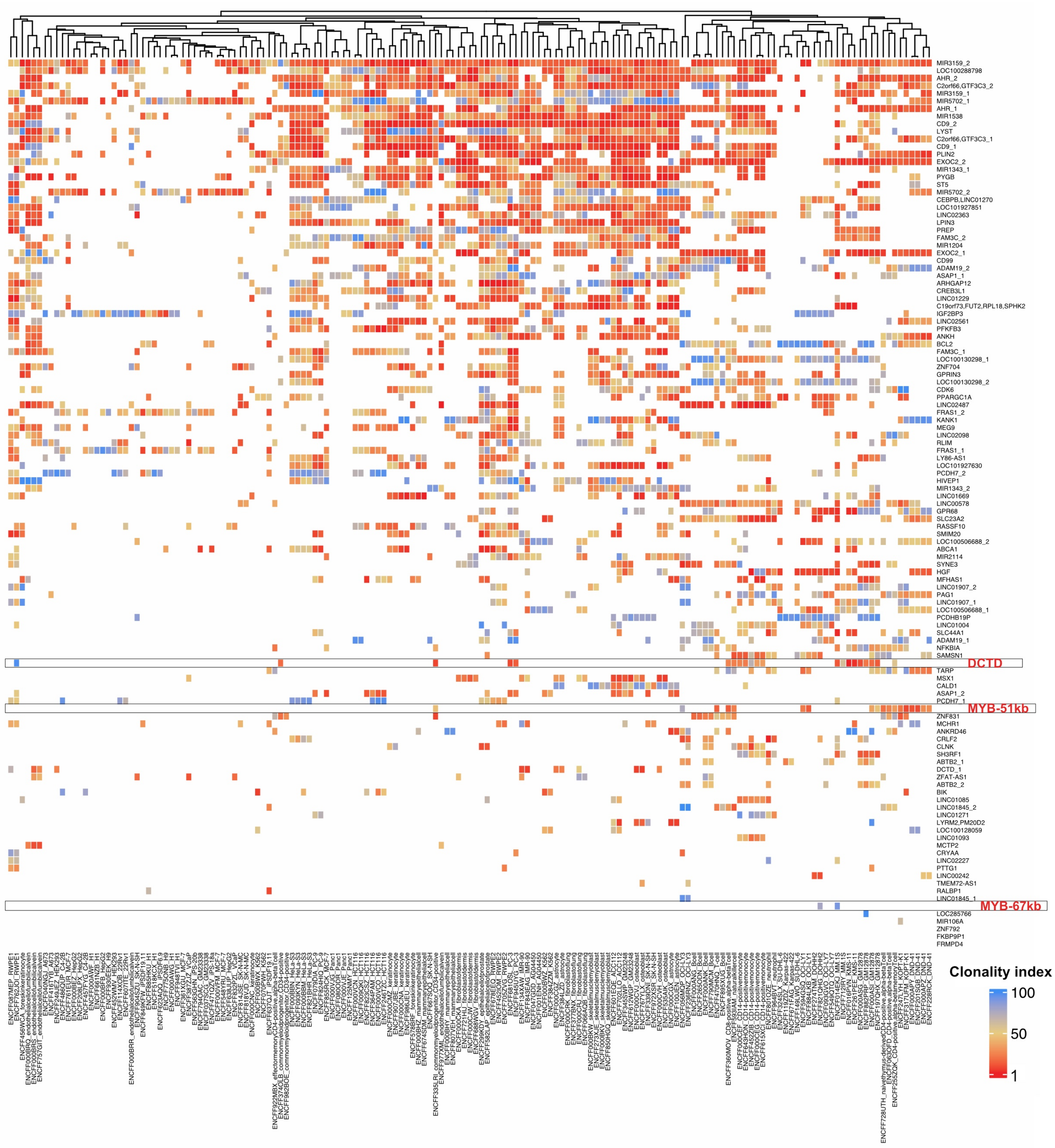

Supplementary Figure 4

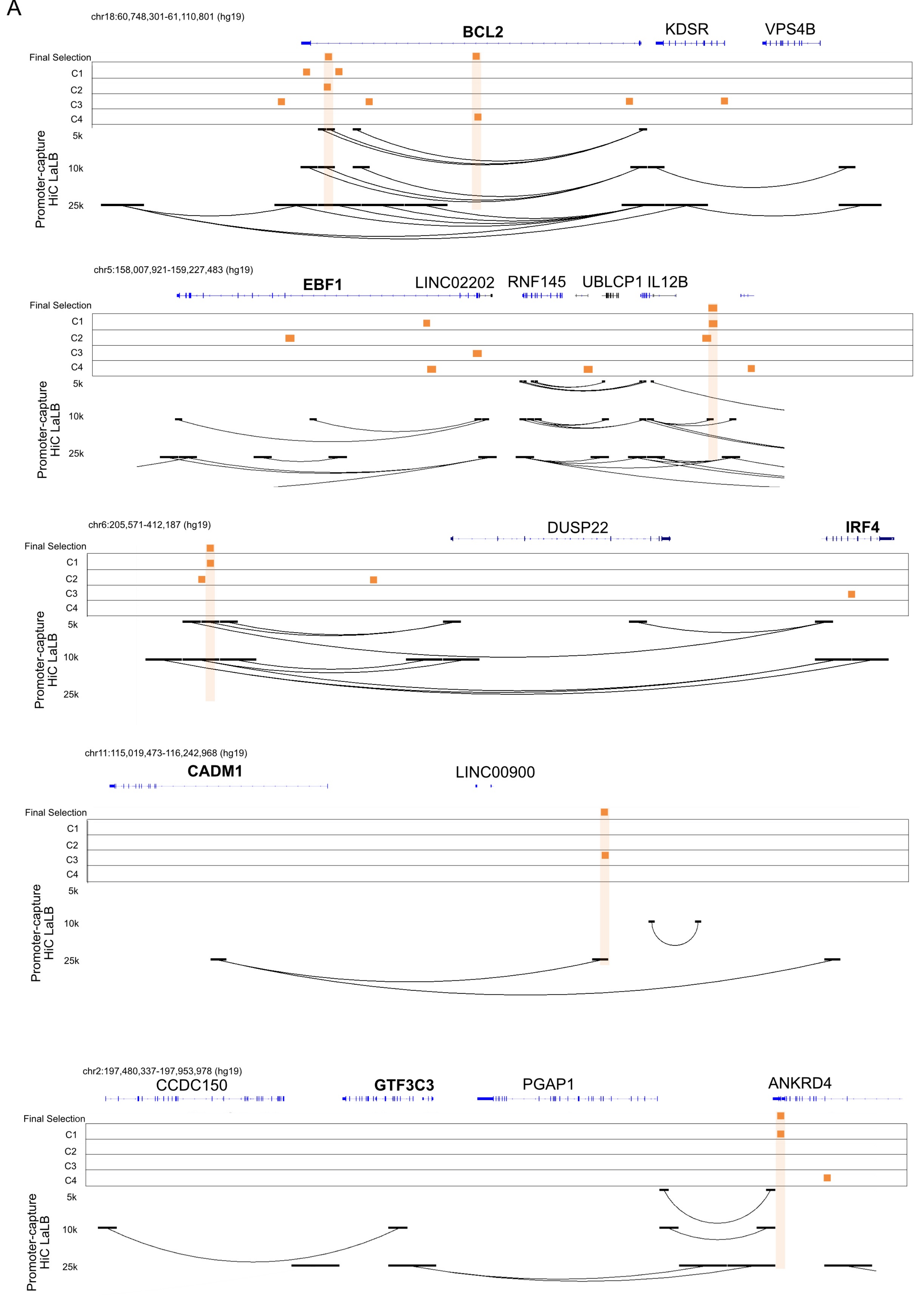

Supplementary Figures 5

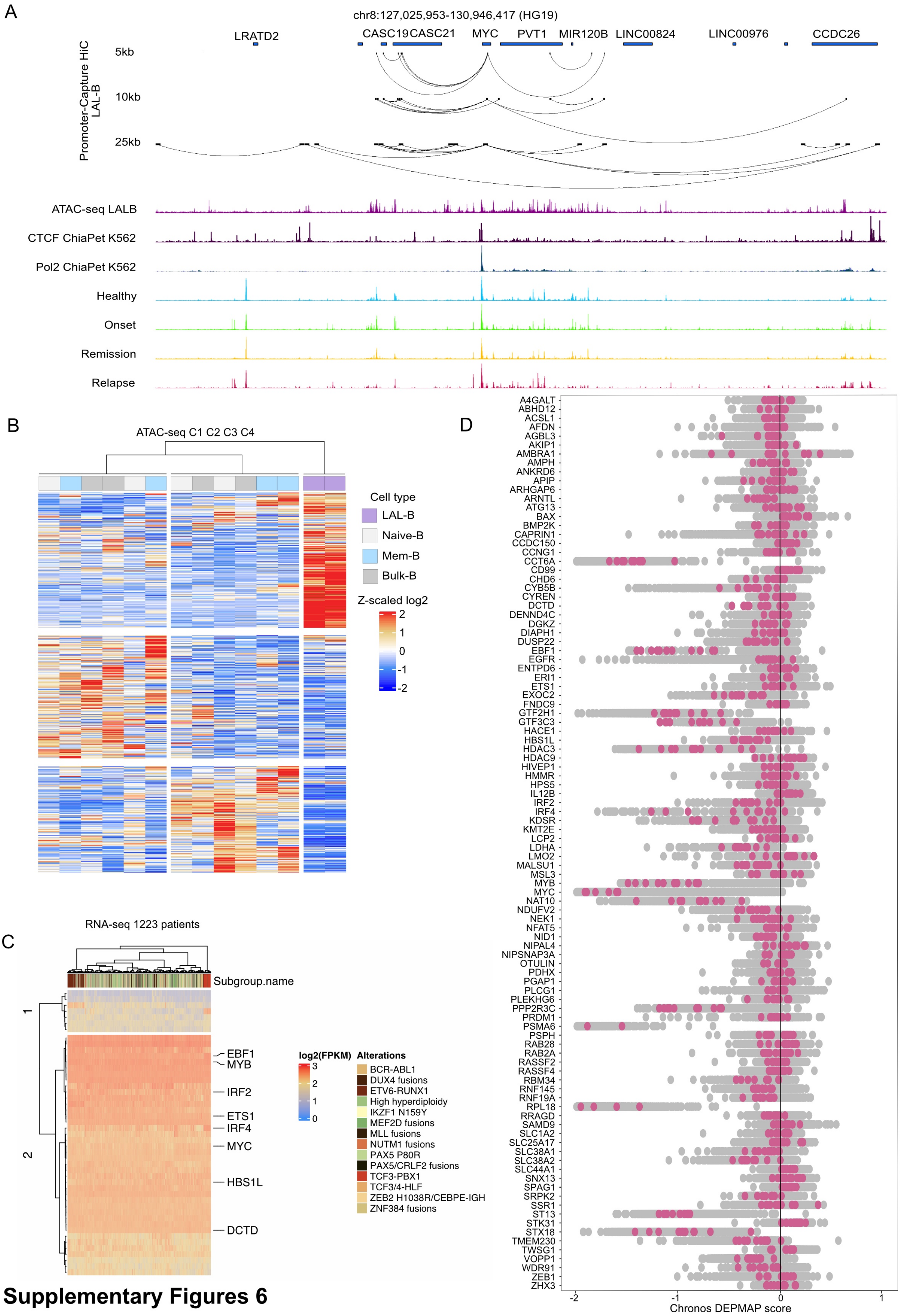

Supplementary Figures 6

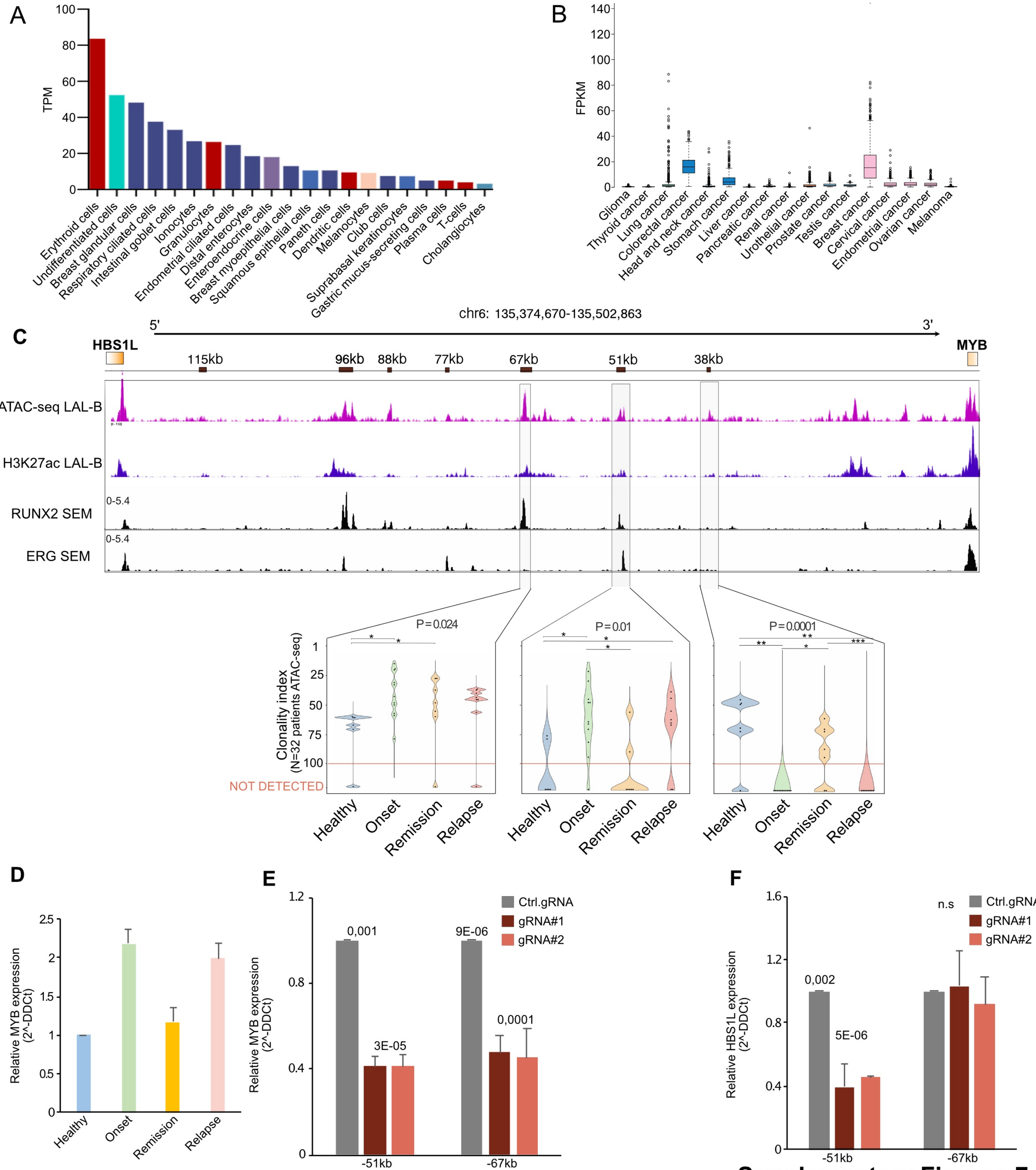

Supplementary Figures 7

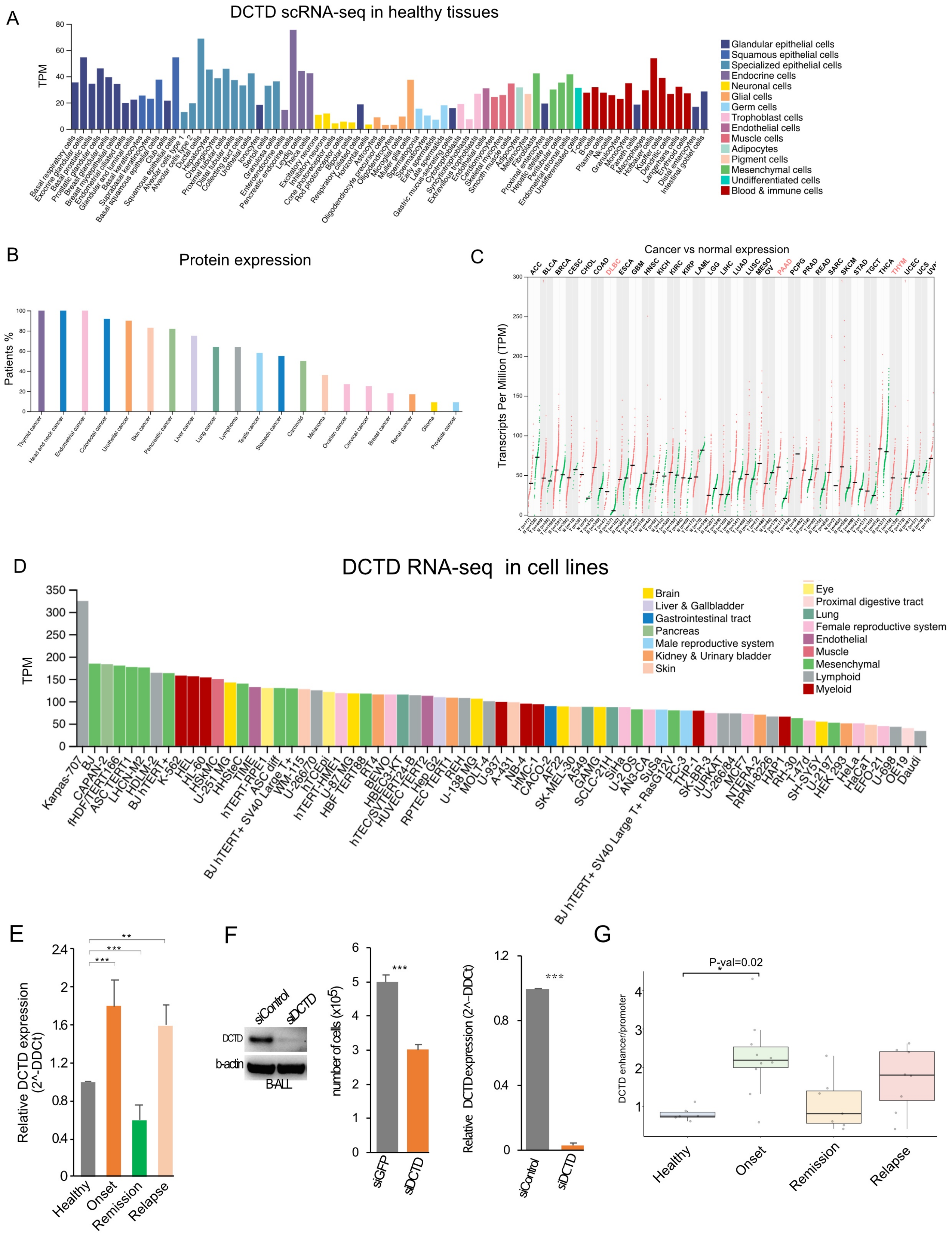

Supplementary Figures 8
